# The Gq/11 family of Gα subunits is necessary and sufficient for lower jaw development

**DOI:** 10.1242/dev.204396

**Published:** 2025-04-17

**Authors:** Stanley M. Kanai, Chloe R. Garcia, MaCalia R. Augustus, Shujan A. Sharafeldeen, Elliott P. Brooks, Juliana Sucharov, Ezra S. Lencer, James T. Nichols, David E. Clouthier

**Affiliations:** ^1^ Department of Craniofacial Biology, School of Dental Medicine, University of Colorado Anschutz Medical Campus, Aurora, CO 80108, USA; ^2^ Department of Biology, Lafayette College, Easton, PA 18042, USA

**Keywords:** Heterotrimeric G protein, Craniofacial development, CRISPR/Cas9, Single cell RNA-sequencing, Small molecule inhibitor, Zebrafish

## Abstract

Vertebrate jaw development is coordinated by highly conserved ligand-receptor systems such as the peptide ligand Endothelin 1 (Edn1) and Endothelin receptor type A (Ednra), which are required for patterning of lower jaw structures. The Edn1/Ednra signaling pathway establishes the identity of lower jaw progenitor cells by regulating expression of numerous patterning genes, but the intracellular signaling mechanisms linking receptor activation to gene regulation remain poorly understood. As a first step towards elucidating this mechanism, we examined the function of the Gq/11 family of Gα subunits in zebrafish using pharmacological inhibition and genetic ablation of Gq/11 activity, and transgenic induction of a constitutively active Gq protein in *edn1^−/−^* embryos. Genetic loss of Gq/11 activity fully recapitulated the *edn1^−/−^* phenotype, with genes encoding G11 being most essential. Furthermore, inducing Gq activity in *edn1^−/−^* embryos not only restored Edn1/Ednra-dependent jaw structures and gene expression signatures but also caused homeosis of the upper jaw structure into a lower jaw-like structure. These results indicate that Gq/11 is necessary and sufficient to mediate the lower jaw patterning mechanism for Ednra in zebrafish.

## Teaser

**Summary:** Genetic and pharmacological manipulation reveal that Gq/11 is a signaling mediator acting downstream of Endothelin 1 and Endothelin Receptor Type A that drives tissue patterning for all lower jaw structures in zebrafish.

## INTRODUCTION

Endothelin A receptor (Ednra) and peptide ligand Endothelin 1 (Edn1) are essential for patterning the embryonic tissue that gives rise to lower jaw structures (Clouthier et al., 1998; Yanagisawa et al., 1998b; Miller et al., 2000; Ozeki et al., 2004). During development, Ednra is expressed in migratory and post-migratory cranial neural crest cells (NCCs) (Kurihara et al., 1995; Clouthier et al., 1998; Yanagisawa et al., 1998a; Miller et al., 2000; Nair et al., 2007; Ruest and Clouthier, 2009), which are multipotent progenitors that give rise to all bone and cartilage structures of the face (Le Douarin and Kalcheim, 1999; Chai et al., 2000; Tang and Bronner, 2020). Post-migratory cranial NCCs in pharyngeal arch 1 (or the mandibular portion of arch 1 in mammals) are stimulated by Edn1 secreted by the adjacent ventral ectoderm (Clouthier et al., 1998; Yanagisawa et al., 1998a; Miller et al., 2000), inducing gene expression that establishes two distinct patterning domains for lower jaw structures: a ventral patterning domain that gives rise to the mandible and Meckel's cartilage and an intermediate patterning domain that gives rise to the jaw joint in zebrafish and middle ear structures in mammals (Clouthier et al., 2000; Miller et al., 2003; Ozeki et al., 2004; Jeong et al., 2008; Ruest and Clouthier, 2009; Talbot et al., 2010; Barske et al., 2016; Askary et al., 2017; Tavares et al., 2017). Edn1/Ednra also antagonizes the effects of Nr2f nuclear receptors and Jagged/Notch, two factors that establish upper jaw identity of cranial NCCs in the dorsal domain of the first and second arches (Zuniga et al., 2010; Barske et al., 2016, 2018; Teng et al., 2017). Thus, Edn1/Ednra plays the dual role of establishing lower jaw identity of cranial NCCs while also establishing the boundary separating the lower jaw and upper jaw patterning domains.

In mice and zebrafish, attenuation of the Edn1/Ednra signaling pathway diminishes intermediate and ventral patterning gene expression, causing expansion of dorsal patterning gene expression into the intermediate and ventral domains. This results in a homeotic transformation of lower jaw structures into upper jaw-like structures (Miller et al., 2000; Ozeki et al., 2004; Ruest and Clouthier, 2009; Pritchard et al., 2020; Kanai et al., 2022). Conversely, ectopic activation of the Edn1/Ednra signaling pathway in the dorsal patterning domain (or the maxillary portion of arch 1 in mammals) results in homeotic transformation of upper jaw structures into lower jaw-like structures (Sato et al., 2008b; Alexander et al., 2011; Zuniga et al., 2011; Tavares and Clouthier, 2015; Barske et al., 2018; Kurihara et al., 2023). In either case in which Edn1/Ednra signaling is lost or ectopically activated, misexpression of patterning genes that are positively or negatively regulated by Edn1/Ednra changes the positional identities of cranial NCCs. While the Edn1/Ednra-regulated genes have been extensively characterized, the intracellular signaling events connecting Ednra activation to gene regulation are not fully understood.

Ednra signaling is mediated by heterotrimeric G proteins, complexes composed of a Gα subunit and a Gβ/Gγ obligate heterodimer (Gβγ) (Gilman, 1987). Ligand-bound Ednra facilitates the exchange of GDP for GTP on the Gα subunit, causing Gα and Gβγ to dissociate and interact with their respective signaling effectors (Oldham and Hamm, 2008). Ednra can couple with all four Gα subunit family members – Gq/11, G12/13, Gs and Gi/o – to varying extents depending on the cell and tissue type (Aramori and Nakanishi, 1992; Khac et al., 1994; Kawanabe et al., 2002; Wettschureck and Offermanns, 2005; Inoue et al., 2019; Okashah et al., 2019; Masuho et al., 2023). In mice, Gq and G11 proteins are encoded by *Gnaq* and *Gna11*, respectively. Removal of *Gnaq* and *Gna11* using conventional knockout alleles results in midgestational lethality prior to the development of craniofacial structures, although embryos with one wild-type allele for *Gnaq* (*Gnaq^+/−^;Gna11^−/−^*) or *Gna11* (*Gnaq^−/−^;Gna11^+/−^*) can survive to term (Offermanns et al., 1998). Notably, *Gnaq^−/−^;Gna11^+/−^* embryos exhibit defects that appear to be confined to the jaw joint and middle ear structures, while *Gnaq^+/−^;Gna11^−/−^* embryos display no craniofacial defects (Offermanns et al., 1998). This suggests that, although both *Gnaq* and *Gna11* are required for early development, *Gnaq* is specifically required for patterning the intermediate domain. Similarly, embryos with a combination of conventional knockout alleles for *Gna11* and NCC-specific conditional knockout alleles for *Gnaq* (*Gnaq^flox/flox^;Gna11^−/−^;*P0-Cre) exhibited craniofacial defects that appeared to be confined to proximal lower jaw structures, including fusion of the jaw joint and hypoplasia of the proximal mandible, tympanic ring, malleus and incus (Dettlaff-Swiercz et al., 2005). In contrast, the entire mandible undergoes a homeotic transformation into a maxilla-like structure in mice lacking *Edn1*, *Ednra* or *Ece1*, a gene that encodes an essential enzyme in the Edn1 biosynthetic pathway (Yanagisawa et al., 1998b; Ozeki et al., 2004; Ruest and Clouthier, 2009). These differences suggest that there are Gq/11-dependent and -independent subdomains within the mandibular arch, with patterning of the Gq/11-independent domain mediated by a different Gα family member (Sato et al., 2008a). Arguing against this model, mice homozygous for knockout alleles of Gs, G12/13 or Gi/o do not exhibit patterning defects in distal lower jaw structures (Jiang et al., 1998; Dettlaff-Swiercz et al., 2005; Wettschureck and Offermanns, 2005; Plummer et al., 2012; Lei et al., 2016). Thus, the question remains whether patterning is mediated solely by Gq/11 or a combination of Gq/11 and other Gα family members.

This question has direct significance for human health. Perturbations to the Ednra signaling pathway are the underlying causes of congenital syndromic disorders, such as mandibulofacial dysostosis with alopecia (Gordon et al., 2015; Kurihara et al., 2023), oro-oto-cardiac syndrome (Pritchard et al., 2020) and auriculo-condylar syndrome (Rieder et al., 2012; Gordon et al., 2013; Marivin et al., 2016; Kanai et al., 2022). Thus, clarification of the G protein family or families mediating Edn1/Ednra-dependent jaw patterning could help elucidate additional signaling pathway components that are linked to human craniofacial differences.

In this study, we examined the role of Gq/11 in craniofacial patterning using multiple orthogonal approaches in zebrafish. Using a Gq/11 small molecule inhibitor YM-254890 (YM) (Takasaki et al., 2004; Nishimura et al., 2010), we demonstrate that YM-treated zebrafish embryos exhibit craniofacial phenotypes and gene expression changes indicative of Ednra signaling attenuation. In addition, we created mutant alleles for three genes that encode zebrafish Gq/11 proteins, *gnaq*, *gna11a* and *gna11b*, and show that triple homozygous mutant larvae exhibit craniofacial phenotypes nearly identical to those observed in *edn1^−/−^* mutant larvae. Furthermore, the craniofacial phenotypes in *edn1^−/−^* mutant embryos can be rescued by inducing expression of a constitutively active form of Gq during craniofacial patterning. Taken together, these results suggest that Gq/11 is necessary and sufficient for establishing intermediate and ventral patterning domains and the subsequent development of all Ednra-dependent skeletal elements of the lower jaw in zebrafish.

## RESULTS

### YM causes craniofacial phenotypes resembling partial loss of Edn1/Ednra signaling

**Fig. 1.**
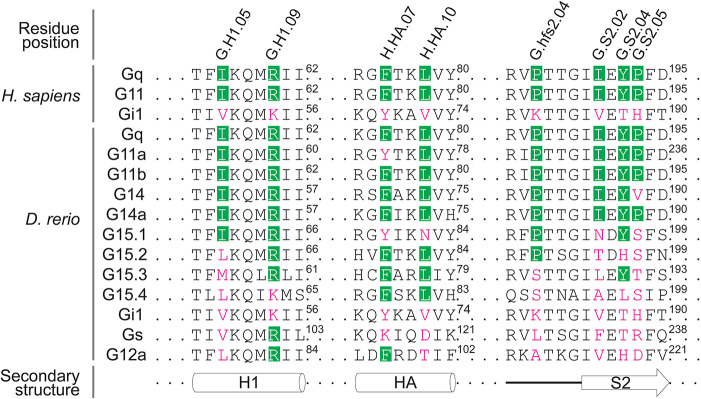
**Sensitivity to YM is determined by eight conserved residues in a subset of Gq/11 family members.** In zebrafish, Gq/11 family members are encoded by *gnaq* (Gq), *gna11a* (G11a), *gna11b* (G11b), *gna14* (G14), *gna14a* (G14a) and *gna15.1-15.4* (G15.1-15.4). Residue positions in Gq/11 family members that interact with YM are annotated with the Common Gα Numbering (CGN) system (Flock et al., 2015). YM-binding residues that are conserved in a subset of Gq/11 family members are shown in green boxes, while diverged residues are labeled in magenta. Superscript numbers indicate the amino acid position for the respective Gα protein. H1 and HA are α-helices of the Ras-like domain and the α helical domain, respectively, and S2 is a β-sheet of the Ras-like domain. These structures constitute the hydrophobic binding pocket for YM in a subset of Gq/11 family members.

To begin our analysis of the G protein signaling pathway downstream of Ednra in zebrafish craniofacial development, we examined the effect of treating zebrafish embryos with YM, a naturally derived compound from *Chromobacterium* species that inhibits a subset of Gq/11 proteins with high selectivity and potency (Takasaki et al., 2004; Nishimura et al., 2010). YM binds to a hydrophobic cleft in mammalian Gq in a region spanning the GTPase (Ras-like) and α-helical domains, stabilizing the allosteric mechanism that promotes GDP release, the rate-limiting step for Gα activation (Nishimura et al., 2010; Flock et al., 2015). This inhibitory effect is dependent on a network of hydrophobic interactions between YM and eight key residues that are present in Gq, G11 and G14, but not in G15 or other Gα family members (Fig. 1) (Nishimura et al., 2010; Onken et al., 2018). In zebrafish, these eight residues are highly conserved in Gq, G11 and G14, weakly conserved in G15 and highly divergent in other Gα family members (Fig. 1), suggesting that YM should display highly specific inhibitory effects towards the Gq/11 signaling pathway in zebrafish, except for G15. Because the expression of G15 is restricted to sensory cells (Ohmoto et al., 2011; Oka and Korsching, 2011), it is unlikely that G15 signaling activity would interfere with our analysis of craniofacial development.

**Fig. 2.**
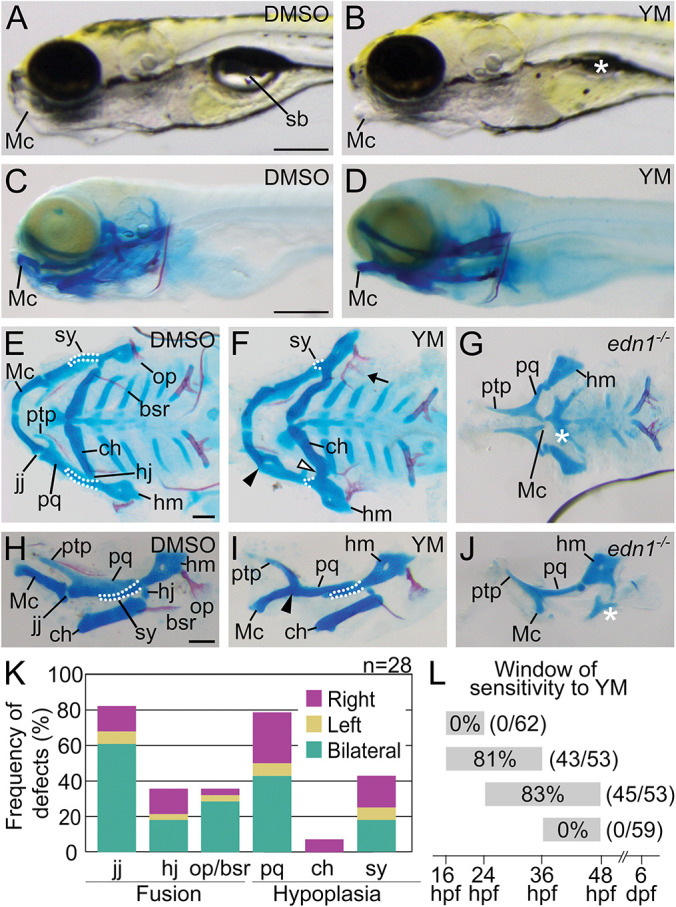
**YM causes defects to a subset of Edn1/Ednra-dependent lower jaw skeletal elements.** (A-D) Lateral views of 6 dpf embryos treated with DMSO (A,C) or YM (B,D), shown in gross (A,B) or whole-mount skeletal preparations of larvae (C,D). The asterisk in B indicates the absence of an inflated swim bladder (sb). (E-J) Ventral (E-G) or lateral (H-J) views of flat-mounts for the viscerocranium from 6 dpf wild-type larvae treated with DMSO (E,H) or YM (F,I), or an untreated *edn1^−/−^* larvae (G,J). In E,F,H,I, white outlines highlight the symplectic cartilage (sy). In F,I, black arrowheads indicate fusion of the jaw joint (jj), white arrowhead indicates fusion of the hyomandibular joint (hj) and the arrow indicates fusion of the opercle (op) and branchiostegal ray (bsr). In G,J, the white asterisk indicates absence of the ceratohyal (ch). (K) Frequency of defects in six Edn1/Ednra-dependent skeletal elements in larvae treated with YM from 16 to 36 hpf. Absence of structure, hypoplasia or fusion were scored as defects (accounting for sidedness). A total of 28 larvae were examined across two independent experiments. The effect of YM was determined to be statistically significant with a chi-square test (*P*=0.0001), comparing the number of defects in DMSO-treated larvae (0) to YM-treated larvae. (L) A schematic illustrating the four overlapping time intervals of YM application (gray bars). Numbers within bars represent the percentages of larvae exhibiting at least one defect to a Edn1/Ednra-dependent lower jaw structure. The total number of larvae examined is given in parentheses. Scale bars: 500 μm (A,C); 100 μm (E,H). hm, hyomandibular; Mc, Meckel's cartilage; pq, palatoquadrate; ptp, pterygoid process of the palatoquadrate.

To test whether YM treatment would produce craniofacial phenotypes, wild-type embryos were incubated with YM or solvent control [dimethyl sulfoxide (DMSO)] from 16 to 36 h post-fertilization (hpf), the approximate window for Edn1/Ednra-dependent craniofacial patterning (Miller et al., 2000, 2003; Walker et al., 2006; Kimmel et al., 2007; Ruest and Clouthier, 2009; Vieux-Rochas et al., 2010; Alexander et al., 2011; Zuniga et al., 2011; Barske et al., 2016, 2018; Meinecke et al., 2018), and then grown to 6 days post-fertilization (dpf) and processed for bone and cartilage staining. We tested a wide range of YM concentrations, from 1 nM to 100 µM. To ensure that the DMSO concentration in the embryo media did not exceed 1% (the upper limit for DMSO that does not cause developmental defects in zebrafish; Hoyberghs et al., 2021), 100 μM YM was the highest concentration we could use (see also Materials and Methods). Compared to control embryos (Fig. 2A,C,E,H), only 100 μM YM produced craniofacial phenotypes, which included an open mouth and uninflated swim bladder (Fig. 2B,D), all indications of defective jaw function (Neuhauss et al., 1996). Additional Edn1/Ednra pathway-associated defects were further visible in flat-mounted preparations of dissected viscerocrania, including hypoplasia of the palatoquadrate and symplectic cartilages, fusion of the jaw and hyomandibular joints, and fusion of the opercle and branchiostegal ray dermal bones (Fig. 2F,I). Defects to at least one of these structures were found in 93% (26/28) of wild-type larvae treated with YM, with considerable variation in the laterality and number of skeletal elements affected per larvae (Fig. 2K).

To determine whether the phenotypes resulting from YM treatment represented a partial or complete loss of Edn1/Ednra signaling, we first generated a new *edn1* allele (*edn1*^*co3009*^) lacking the majority of the coding sequence for the Edn1 bioactive peptide (Fig. S1). The original *edn1* mutant allele *sucker* results from an amino acid substitution (p.Asp8Val) in the bioactive portion of Edn1 (Miller et al., 2000) (Fig. S1). While amino acid substitutions at p.Asp8 (e.g. p.Asp8Asn, p.Asp8Ala) result in approximately 99% less vasoconstrictor activity than that observed for wild-type Edn1 *in vitro* (Huggins et al., 1993), the activity of Edn1 encoded by the *sucker* allele has never been formally tested. Larvae homozygous for the *edn1*^*co3009*^ allele exhibited absence and severe hypoplasia of ventral structures (Fig. 2G,J) and recapitulated the *sucker* craniofacial phenotype (Fig. S1), indicating that the *sucker* and *edn1*^*co3009*^ craniofacial phenotypes are caused by a complete loss of Edn1 activity. In contrast, the phenotypes of YM-treated larvae more closely resembled phenotypes caused by partial loss of Edn1/Ednra signaling (Walker et al., 2006; Kimmel et al., 2007; Miller et al., 2007; Talbot et al., 2010). These data suggest that YM-induced defects are caused by partial inhibition of the Edn1/Ednra signaling pathway.

To determine the developmental time during which Gq/11 activity is required for lower jaw development, embryos were treated with 100 µM YM across four overlapping time intervals (16-24, 16-36, 24-48 or 36-48 hpf) and then analyzed for craniofacial skeleton defects at 6 dpf. Craniofacial defects were observed only in embryos treated from 16 to 36 hpf, or between 24 and 48 hpf (Fig. 2L), with the percentage of affected larvae (Fig. 2L) and frequency and severity of affected skeletal elements (Fig. S2) being similar for both time intervals. These results suggest that Gq/11 activity is required for patterning between 24 and 36 hpf. This window of sensitivity to YM is similar to the window of sufficiency for Edn1/Ednra signaling in craniofacial patterning in zebrafish and mice (Miller et al., 2000, 2003; Walker et al., 2006; Kimmel et al., 2007; Ruest and Clouthier, 2009; Vieux-Rochas et al., 2010; Alexander et al., 2011; Zuniga et al., 2011; Barske et al., 2016, 2018; Meinecke et al., 2018).

### YM causes gene expression changes in cranial NCCs that resemble partial loss of Edn1/Ednra signaling

**Fig. 3.**
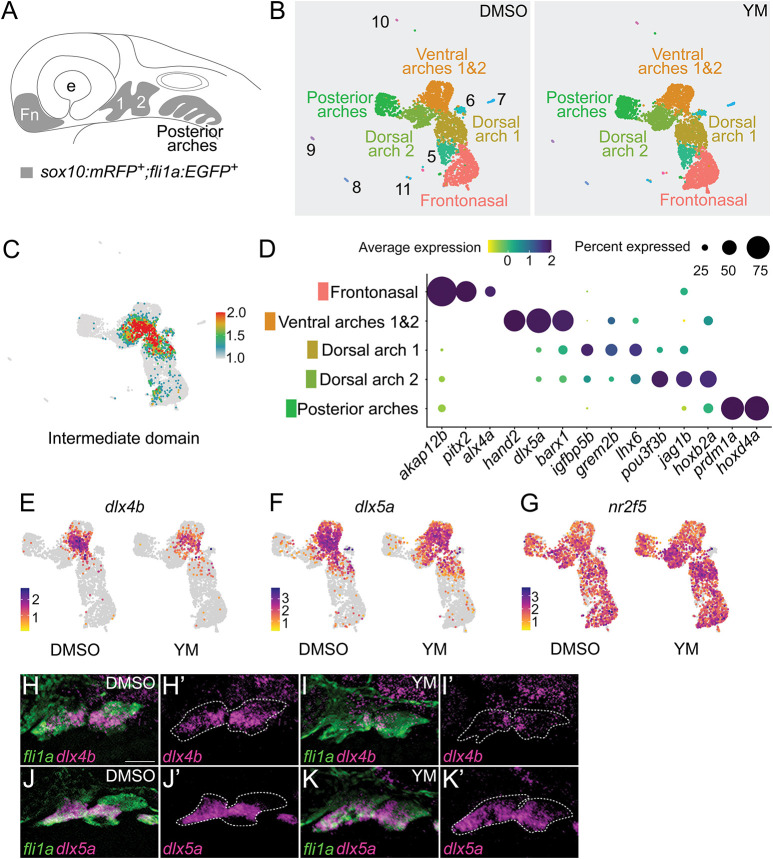
**YM reduces expression of intermediate patterning genes and increases expression of dorsal patterning genes.** (A) Schematic of a zebrafish embryo at 36 hpf. Cells double labeled with *sox10:mRFP* and *fli1a:EGFP* transgenic reporters (highlighted in gray) represent cranial neural crest populations from the frontonasal region (Fn), anterior pharyngeal arches 1 (1) and 2 (2), and posterior pharyngeal arches. (B) UMAP plots for DMSO or YM-treated samples. Clusters analyzed in this study are labeled with the NCC populations they represent. Clusters 5-11 are described further in Fig. S3 and Table S1. Equivalent clusters between DMSO and YM-treated samples are labeled with the same colors. (C) Feature map highlighting approximate cell populations in the intermediate domains of pharyngeal arches 1 and 2, shown overlaid on combined UMAP plots of control and YM-treated samples. The feature map represents the composite average expression level for 14 experimentally verified intermediate domain patterning genes: *ccn2b*, *dlx3b*, *dlx4a*, *dlx4b*, *emx2*, *fgfbp2a*, *foxc1b*, *foxd1*, *fsta*, *grem2b*, *igfbp5b*, *msx1a*, *nkx3-2* and *shox* (see also Fig. S4). The scale is average expression. (D) Dot plot of selected marker genes and their respective cluster identity (Fig. S3). (E-G) Feature maps highlighting differential expression of *dlx4b* (E), *dlx5a* (F) and *nr2f5* (G) in DMSO or YM-treated samples. The scale is average expression. (H-K′) Fluorescence *in situ* hybridization and immunofluorescence of 36 hpf embryos treated with DMSO or YM from 16 to 36 hpf. DMSO (H,H′,J,J′) or YM-treated (I,I′,K,K′) embryos were probed for *dlx4b* (H-I′) or *dlx5a* (J-K′) using fluorescence *in situ* hybridization (magenta). Pharyngeal arches, labeled with *fli1a:EGFP*, were detected with immunofluorescence (green)*.* Approximate borders for pharyngeal arches 1 and 2 are indicated with dashed lines (H′,I′,J′,K′). Images are representative of four embryos. Scale bar: 50 μm.

The loss of Edn1/Ednra signaling causes characteristic changes to gene expression in NCCs of the first and second pharyngeal arches, including downregulation of lower jaw patterning genes and upregulation of upper jaw patterning genes in the intermediate and ventral domains (Clouthier et al., 1998, 2000; Thomas et al., 1998; Miller et al., 2000, 2003; Ozeki et al., 2004; Ruest et al., 2004; Walker et al., 2006, 2007; Nair et al., 2007; Sato et al., 2008a; Tavares et al., 2012, 2017; Barske et al., 2016, 2018; Askary et al., 2017). To determine whether YM caused similar changes to gene expression in cranial NCCs as does partial loss of Edn1/Ednra signaling, we performed single-cell RNA sequencing (scRNA-seq) on cranial NCCs isolated from *Tg(sox10:mRFP;fli1a:EGFP)* embryos treated with DMSO or 100 µM YM from 16 to 36 hpf (Fig. 3A). Fluorescence-activated cell sorting (FACS) of *sox10:mRFP;fli1a:EGFP* double-positive cells allows purification of cranial NCC populations in zebrafish embryos (Askary et al., 2017; Mitchell et al., 2021). Subsequent analyses with Seurat identified 12 cell clusters with unique transcriptional profiles (Fig. 3B, Fig. S3, Table S1). Using known marker genes (Miller et al., 2003; Askary et al., 2017; Barske et al., 2018; Mitchell et al., 2021; Fabian et al., 2022; Stenzel et al., 2022), NCC populations were identified from the frontonasal region (*akap12b^+^*, *alx4a^+^*), anterior pharyngeal arches 1 and 2 (*dlx2a^+^*, *dlx5a^+^*) and posterior pharyngeal arches 3-7 (*prdm1a^+^*) (Fig. 3B,D). The NCCs of pharyngeal arches 1 and 2 were distributed across three clusters, with one cluster representing the ventral domain cells of both arches 1 (*hand2^+^*) and 2 (*hand2^+^*, *hoxb2a^+^*), and two separate clusters representing dorsal domain cells of arch 1 *(pou3f3b^+^*, Hox−) and arch 2 (*jag1b^+^*, *hoxb2a^+^*). Although we did not find a dedicated cluster for intermediate domain cells, we used known marker genes to identify the approximate population of intermediate domain cells nested within the ventral and dorsal clusters (Fig. 3C, Fig. S4). The remaining clusters appeared to consist of NCC-derived pigment cells (cluster 11: *dct*^+^, *tyrp1b*^+^) and hematopoietic derivatives (cluster 9: *hbbe3^+^*, *hbae3^+^*; cluster 10: *myb^+^*, *lyz^+^*); these clusters were not examined further in this study.

To examine the effects of YM on gene expression, we performed differential expression analysis between equivalent clusters in YM-treated samples relative to control samples (Table S2). Differentially expressed genes with adjusted *P*-values less than 0.05 were found in six out of eleven clusters – frontonasal, ventral arches 1 and 2, dorsal arch 1, dorsal arch 2, posterior arches 3-7 and cluster 5 (unknown identity) (Fig. S5, Table S2). Overall, a total of 36 and 34 unique, non-overlapping genes were upregulated or downregulated, respectively, in YM-treated samples. Of the aberrantly upregulated genes, dorsal patterning genes including, *hey1*, *her6*, *her9* and *nr2f5*, were present in the ventral and dorsal arch clusters (Fig. 3G, Fig. S5, Table S2) (Zuniga et al., 2010; Barske et al., 2016, 2018; Askary et al., 2017). The majority of aberrantly downregulated genes were in the ventral arches 1 and 2 and dorsal arch 1. In the ventral arch cluster, more than half of the reduced genes were Ednra targets. Notably, the intermediate patterning genes *dlx4a* and *dlx4b* (Fig. 3E,H-I′, Fig. S5) were downregulated to a greater extent than ventral patterning genes such as *hand2* and *dlx5a* (Fig. 3F,J-K′, Fig. S5). These gene expression changes are similar to changes observed in animal models with partial loss of Edn1/Ednra signaling (Walker et al., 2006, 2007; Miller et al., 2007; Sato et al., 2008a; Ruest and Clouthier, 2009; Tavares et al., 2012). Furthermore, the relatively modest downregulation of ventral patterning genes compared with intermediate patterning is consistent with the unperturbed ventral domain-derived skeletal structures in YM-treated larvae (Fig. 2F,I). These results indicate that YM partially attenuates the Edn1/Ednra signaling pathway in cranial NCCs through the inhibition of Gq/11.

### Pharmacogenetic interactions suggest that Gq/11 is also required for ventral domain patterning

**Fig. 4.**
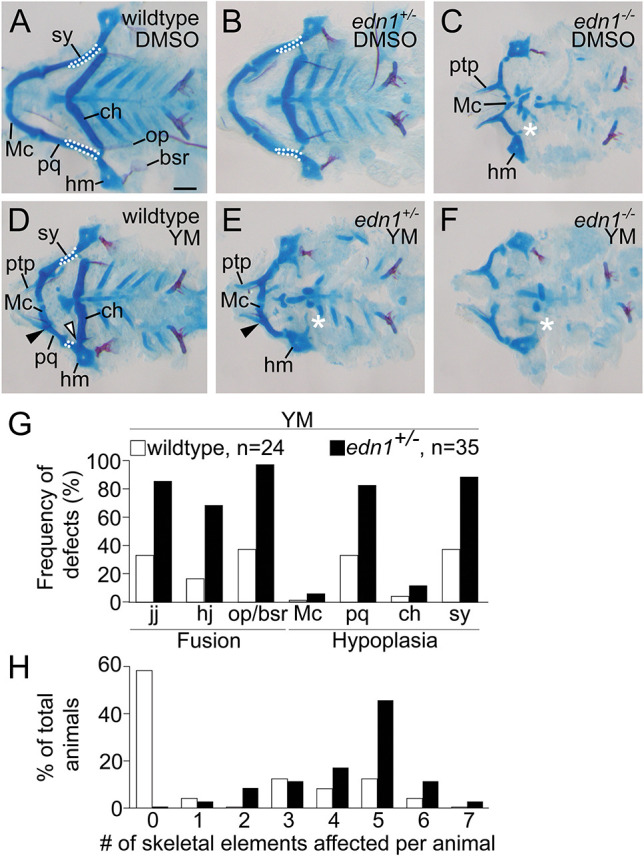
**YM increases the prevalence and severity of lower jaw defects in *edn1^+/−^* larvae relative to wild-type larvae.** (A-F) Representative flat-mounts of the viscerocranium from wild-type (A,D), *edn1^+/−^* (B,E) and *edn1^−/−^* (C,F) larvae treated with DMSO (A-C) or 100 μM YM (D-F) from 16 to 36 hpf. In A,B,D, white outlines highlight the symplectic cartilage (sy). In C,E,F, white asterisks indicate absence of the ceratohyal (ch). In D,E, black arrowheads indicate fusion of the jaw joint (jj). In D, the white arrowhead indicates fusion of the hyomandibular joint (hj). All skeletal preparations are 6 dpf. (G) Frequency of defects in seven Edn1/Ednra-dependent skeletal elements in YM-treated wild-type or *edn1^+/−^* larvae. Absence of structure, hypoplasia or fusion were scored as defects (ignoring sidedness). The pharmacogenetic interaction between *edn1* and YM was determined to be statistically significant with a chi-square test (*P*=0.0001), comparing the number of defects in YM-treated *edn1^+/+^* and *edn1^+/−^* larvae. (H) Percentage of individual larvae presenting with defects in the seven Edn1/Ednra-dependent structures. Individual larvae were scored for total number of skeletal elements affected (ignoring sidedness). Bar graphs are color-coded the same as G. All wild-type, *edn1^+/−^* or *edn1^−/−^* embryos treated with DMSO or YM are siblings from the same clutch. Scale bar: 100 μm. bsr, branchiostegal ray; hm, hyomandibular; Mc, Meckel's cartilage; op, opercle; pq, palatoquadrate; ptp, pterygoid process of the palatoquadrate.

Because we could not use a YM concentration greater than 100 µM, we could not distinguish whether the YM-generated phenotype was due to incomplete inhibition of Gq/11 activity or whether Gq/11 activity was only required in the intermediate domain. To address this question, we tested the effects of YM on *edn1^+/−^* zebrafish embryos, with the supposition that these embryos would have reduced expression of Ednra-dependent genes similar to *Edn1^+/−^* mouse embryos (Vieux-Rochas et al., 2010) and thus would be sensitized to the effects of YM. Embryos generated from *edn1^+/−^* mating pairs were treated with 100 µM YM or DMSO from 16 to 36 hpf, then processed for skeletal preparations at 6 dpf. DMSO treatment did not cause defects to any skeletal elements in either wild-type or *edn1^+/−^* larvae (Fig. 4A,B). In YM-treated wild-type larvae, skeletal defects were confined to intermediate domain structures, with the ventral portion of the Meckel's cartilage and ceratohyal unaffected (Fig. 4D,G). However, in YM-treated *edn1^+/−^* larvae, skeletal defects expanded to include ventral domain structures, shown by hypoplasia of the Meckel's cartilage and ceratohyal (Fig. 4E,G). Further, YM treatment resulted in more skeletal elements affected per individual in *edn1^+/−^* larvae compared to wild-type larvae (Fig. 4H). YM treatment had no effect on the phenotype of *edn1^−/−^* larvae (Fig. 4C,F). The increased severity and frequency of defects elicited by this pharmacogenetic interaction (Fig. 4G,H) suggests that YM can only partially inhibit Gq/11 activity in wild-type zebrafish embryos, whereas the inhibitory effect of YM on Gq/11 activity can be augmented in a *edn1^+/−^* genetic background. These findings suggest that Gq/11 is required for patterning beyond the intermediate domain.

### G11 paralogs are necessary for development of all Edn1/Ednra-dependent craniofacial structures

**Fig. 5.**
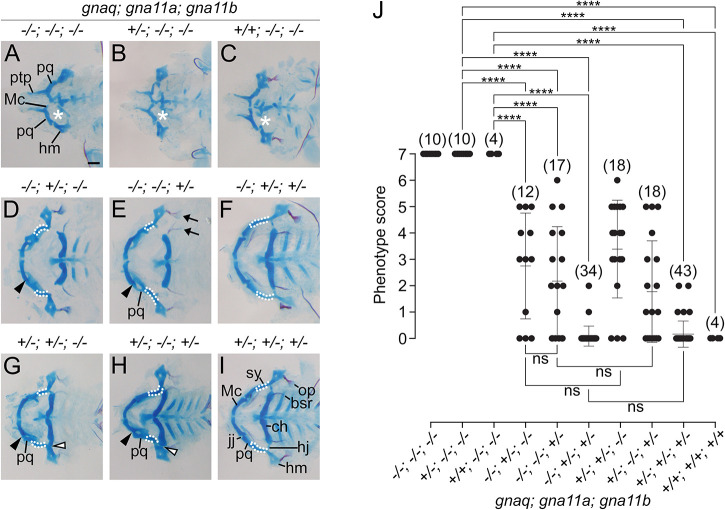
***gna11a a*nd *gna11b*, but not *gnaq*, are necessary for development of Edn1/Ednra-dependent structures.** (A-I) Representative flat-mounts of the viscerocranium at 6 dpf, shown in ventral view, for selected allelic combinations. In D-I, white outlines highlight the symplectic (sy). In A-C, the white asterisks indicate absence of the ceratohyal (ch). In D,E,G,H, black arrowheads indicate fusion of the jaw joint (jj). In G,H, the white arrowheads indicate fusion of the hyomandibular joint (hj). (J) Overall severity of defects for genotypes shown in A-I were quantified as a ‘phenotype score’. Individual larvae were scored for the total number of Edn1/Ednra-dependent skeletal elements exhibiting defects (ignoring sidedness). A phenotype score of 0 corresponds to a wild-type phenotype, and a score of 7 indicates all Edn1/Ednra-dependent structures on at least one side were affected. One dot represents an individual larva. The middle line is the mean. Error bars represent s.e.m. The *n* values for respective genotypes are indicated in parentheses. Statistical significance was determined using one-way ANOVA with Holm–Šídák multiple comparisons test (****P<0.0001; ns, not significant). Scale bar: 100 μm. bsr, branchiostegal ray; hm, hyomandibular; Mc, Meckel's cartilage; op, opercle; pq, palatoquadrate; ptp, pterygoid process of the palatoquadrate.

To interrogate more comprehensively the role of Gq/11 in facial development, we mutated genes encoding Gq and G11 proteins using CRISPR/Cas9. In zebrafish, Gq is encoded by *gnaq*, and G11 is encoded by two paralogs, *gna11a* and *gna11b.* Although *gnaq* expression in the embryonic head was undetectable by scRNA-seq (Fig. S6), quantitative PCR (qPCR; Fig. S7) or colorimetric *in situ* hybridization (Fig. S8), we proceeded to target the *gnaq* allele given the importance of *Gnaq* in mouse lower jaw development (Offermanns et al., 1998). Of the two G11 genes, *gna11b* expression was relatively higher than *gna11a* in scRNA-seq, qPCR and colorimetric *in situ* hybridization analyses (Figs S6-S8). Other Gq/11 family members, including *gna14*, *gna14a* and *gna15.1-15.4*, were not considered because they exhibit cell type-specific expression in sensory organs and hematopoietic lineages (Ohmoto et al., 2011; Oka and Korsching, 2011). We designed CRISPR/Cas9 guide RNAs to target exon 3 or 4 of the genes (Fig. S9A), similar to targeting strategies used for *Gnaq* and *Gna11* knockout alleles in mice (Offermanns et al., 1998). For all three genes, we identified a frame-shift mutation that is predicted to result in a premature stop codon and non-functional protein product (Fig. S9B). Similar to wild-type larvae (Fig. 2), larvae and adults that were homozygous for a single mutant allele (*gnaq^−/−^*, *gna11a^−/−^* or *gna11b^−/−^*) (Fig. S9) or triple heterozygous for all mutant alleles (*gnaq^+/−^;gna11a^+/−^;gna11b^+/−^*) (Fig. 5I) did not exhibit overt craniofacial phenotypes and were viable and fertile. However, we observed a spectrum of craniofacial phenotypes in larvae generated from *gnaq^+/−^;gna11a^+/−^;gna11b^+/−^* mating pairs (Fig. 5). A total of 336 larvae were processed for skeletal preparations at 6 dpf, followed by analysis of genotypes and phenotypes (Table S3). Flat-mounts of skeletal preparations for selected genotypes are shown in Fig. 5A-I. Quantification of the severity and frequency of defects for selected genotypes is shown in Fig. 5J. The most severe defects were observed in *gnaq^−/−^;gna11a^−/−^;gna11b^−/−^* (Fig. 5A,J), *gnaq^+/−^;gna11a^−/−^;gna11b^−/−^* (Fig. 5B,J) and *gnaq^+/+^;gna11a^−/−^;gna11b^−/−^* (Fig. 5C,J) larvae, which exhibited defects in all Edn1/Ednra-dependent skeletal elements and resembled the *edn1^−/−^* phenotype (Fig. 2G,J). This suggests that *gna11a* and *gna11b* are required for zebrafish lower jaw development, but *gnaq* is not. Larvae with one wild-type allele of either *gna11a* (*gnaq^−/−^;gna11a^+/−^;gna11b^−/−^*) (Fig. 5D) or *gna11b* (*gnaq^−/−^;gna11a^−/−^;gna11b^+/−^*) (Fig. 5E) exhibited phenotypes that were all less severe and more variable than that of *gnaq^−/−^;gna11a^−/−^;gna11b^−/−^* larvae (Fig. 5A,J). Larvae with one wild-type allele of *gna11a* and *gna11b* (*gnaq^−/−^;gna11a^+/−^;gna11b^+/−^*) (Fig. 5F) exhibited further reduced phenotypic severity and variation (Fig. 5J). Importantly, the addition of a wild-type *gnaq* allele to the above *gna11a* and *gna11b* allelic combinations – *gnaq^+/−^;gna11a^+/−^;gna11b^−/−^* (Fig. 5G), *gnaq^+/−^;gna11a^−/−^;gna11b^+/−^* (Fig. 5H) and *gnaq^+/−^;gna11a^+/−^;gna11b^+/−^* (Fig. 5I) – had no effect on phenotypic severity and variation (Fig. 5J)*.* Taken together, these results suggest that *gna11a* and *gna11b* are necessary for the development of Edn1/Ednra-dependent craniofacial structures in a gene-dosage dependent manner, whereas *gnaq* appears to be dispensable.

### Gq is sufficient for lower jaw specification

**Fig. 6.**
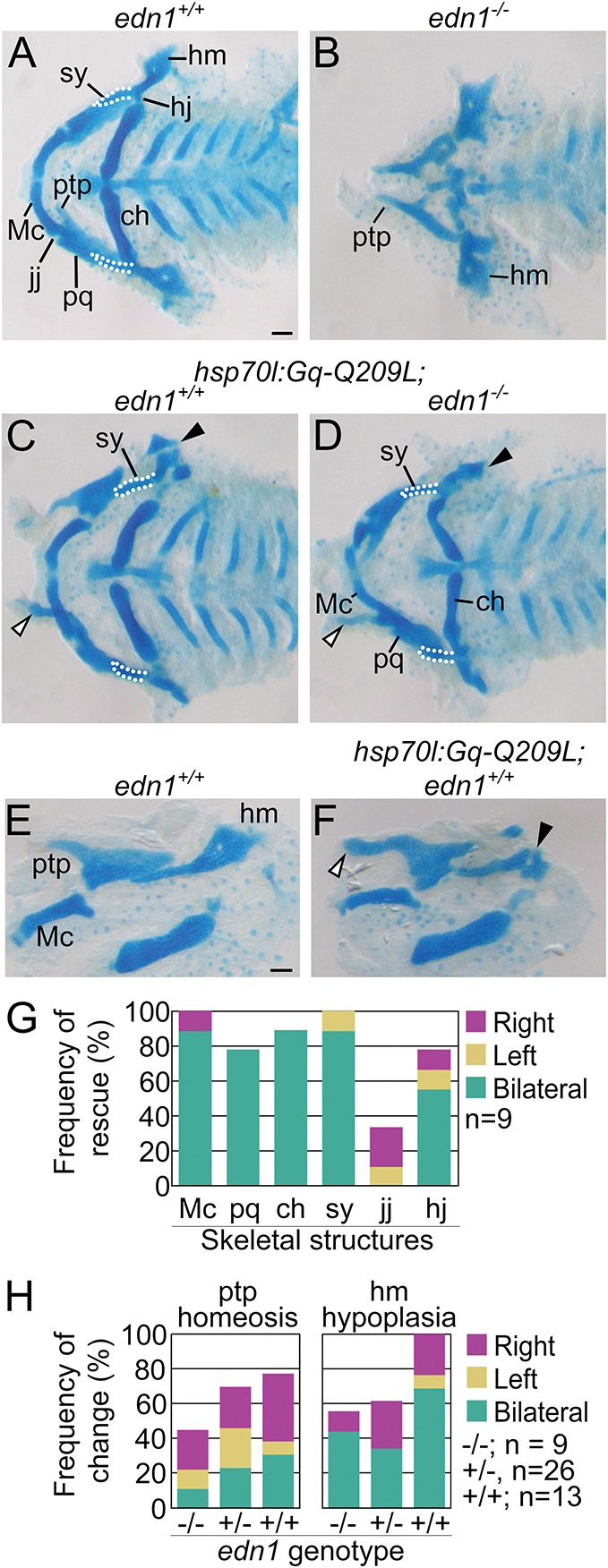
**Induction of Gq activity rescues the *edn1^−/^*^−^ phenotype and causes ventralization of dorsal structures.** (A-D) Representative flat-mounts of the viscerocranium at 4 dpf, shown in ventral view, of heat-shocked larvae for *edn1^+/+^* (A), *edn1^−/−^* (B), *hsp70l:Gq-Q209L;edn1^+/+^* (C) and *hsp70l:Gq-Q209L;edn1^−/−^* (D). Transformed pterygoid process of the palatoquadrate (ptp) is indicated by white arrowheads in C and D. Transformed hyomandibular cartilage (hm) is indicated by a black arrowhead in C,D. (E,F) Flat-mounts of the viscerocranium at 4 dpf, in lateral view, of heat-shocked non-transgenic *edn1^+/+^* (E) and *hsp70l:Gq-Q209L; edn1^+/+^* larvae (F). Transformed ptp (white arrowhead) and hm (black arrowhead) are indicated in F. (G) Frequency of phenotype rescue for six Edn1/Ednra-dependent structures in heat-shocked *hsp70l:Gq-Q209L;edn1^−/−^* larvae (accounting for sidedness). Phenotype rescue was determined to be statistically significant with a chi-square test (*P=*0.0001), comparing the number of restored skeletal elements in non-transgenic *edn1^−/−^* larvae (0) to *hsp70l:Gq-Q209L;edn1^−/−^* larvae. (H) Frequency of malformed ptp and hm (accounting for sidedness) in all *edn1* genotypes with the *hsp70l:Gq-Q209L* transgene. Scale bar: 100 μm. ch, ceratohyal; hj, hyoid joint; jj, jaw joint; Mc, Meckel's cartilage; pq, palatoquadrate; sy, symplectic.

To determine whether Gq/11 signaling activity is sufficient for lower jaw development, we examined whether we could rescue the *edn1^−/−^* mutant phenotype by expressing a constitutively active form of Gq, Gq-Q209L (Fig. 6) (Kalinec et al., 1992). The Q209L amino acid substitution prevents GTP hydrolysis and maintains Gq in an active signaling state independently of receptor activity (Kleuss et al., 1994). Gq and G11 proteins are functionally redundant (Malbon, 2005; Wettschureck and Offermanns, 2005), and the constitutively active mutants Gq-Q209L and G11-Q209L activate the same signaling pathways in the context of uveal melanoma (Annala et al., 2019; Lapadula et al., 2019). In order to control the timing of induction for Gq activity, we made a transgenic line that expresses Gq-Q209L under the regulation of a *hsp70l* heat shock-inducible promoter (Halloran et al., 2000) (*hsp70l:Gq-Q209L*)*.* First, we determined whether heat shock induces Gq-Q209L expression in the craniofacial mesenchyme by examining changes to ERK1/2 (Mapk3/Mapk1) phosphorylation levels, an established readout for Gq/11 activity (Van Raamsdonk et al., 2009). Immunohistochemistry of coronal sections using an antibody for phosphorylated ERK1/2 (Randlett et al., 2015) confirmed that ERK1/2 phosphorylation levels were elevated in the craniofacial mesenchyme of *hsp70l:Gq-Q209L* embryos but not in sibling control embryos (Fig. S10). Next, embryos generated from *edn1^+/−^* and *edn1^+/−^;hsp70l:Gq-Q209L* mating pairs were heat-shocked at 16 hpf and then processed for fluorescence *in situ* hybridization at 28 hpf or skeletal preparations at 4 dpf. We performed skeletal preparations at 4 dpf instead of 6 dpf because a small percentage of *hsp70l:Gq-Q209L-*positive larvae exhibited early embryonic lethality starting at 5 dpf due to substantial cardiac edema (Fig. S11). In *edn1^+/+^* or *edn1^−/−^* larvae (sibling controls), heat shock did not affect development of the craniofacial skeleton (Fig. 6A,B). In heat-shocked *edn1^−/−^;hsp70l:Gq-Q209L* embryos, the majority of Edn1/Ednra-dependent structures were restored in 100% (9/9) of larvae (Fig. 6D,G). The palatoquadrate, symplectic, ceratohyal, Meckel's cartilage and the hyomandibular joint were restored at the highest frequency, whereas jaw joint restoration occurred at a lower frequency (Fig. 6G). We also observed homeotic transformations of upper jaw structures to lower jaw-like structures in heat-shocked *edn1^+/+^;hsp70l:Gq-Q209L* (Fig. 6C,F,H) and *edn1^−/−^;hsp70l:Gq-Q209L* (Fig. 6D,H) embryos. The pterygoid process of the palatoquadrate, typically a short, pointed structure (Fig. 6A,B,E), was transformed into a long, broad piece of cartilage resembling Meckel's cartilage (Fig. 6C,D,F). The dorsal portion of the hyomandibular cartilage was also hypoplastic and misshapen (Fig. 6,D,F). Similar transformations to the pterygoid process and hyomandibular cartilages have been reported in larvae with ectopic Ednra signaling activity in the dorsal domain of the first and second arches (Kimmel et al., 2007; Alexander et al., 2011; Zuniga et al., 2011; Barske et al., 2018).

**Fig. 7.**
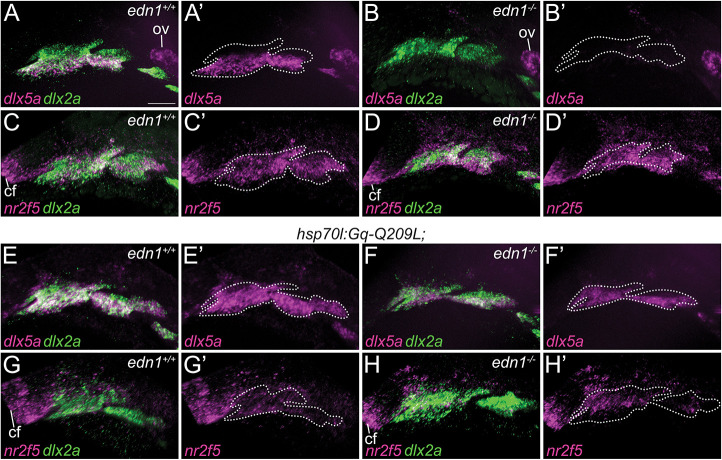
**Induction of Gq activity upregulates *dlx5a* expression and downregulates *nr2f5* expression.** (A-H′) The expression patterns of *dlx5a* and *nr2f5*, both in magenta, are shown by two-color fluorescence *in situ* hybridization on 28 hpf embryos. Pharyngeal arches are labeled, with *dlx2a* in green. *dlx5a* and *dlx2a* (A,B,E,F) or *nr2f5* and *dlx2a* (C,D,G,H) are shown overlaid. *dlx5a* (A′,B′,E′,F′) and *nr2f5* (C′,D′,G′,H′) are also shown alone, with the border of the pharyngeal arches indicated by white dashed lines. (A,A′,C,C′) Expression in non-transgenic *edn1^+/+^* embryos. (E,E′,G,G′) Expression in *hsp70l:Gq-Q209L;edn1^+/+^* embryos. (B,B′,D,D′) Expression in non-transgenic *edn1^−/−^* embryos. (F,F′,H,H′) Expression in *hsp70l:Gq-Q209L;edn1^−/−^* embryos. All embryos were heat-shocked. Images are representative of at least five embryos. Scale bar: 50 μm. cf, choroid fissure; ov, otic vesicle.

These morphological changes were preceded by changes to patterning gene expression (Fig. 7). In 28 hpf *edn1^−/−^* embryos, the expression of *dlx5a*, a transcription factor essential for lower jaw specification, was severely reduced in the first and second pharyngeal arch mesenchyme (Fig. 7B,B′). In *edn1^−/−^;hsp70l:Gq-Q209L* embryos that were heat-shocked, *dlx5a* expression was not only restored in the ventral and intermediate domains of the first and second arches, but also expanded into the dorsal domain (Fig. 7F,F′). Similar expansion of *dlx5a* was observed in *edn1^+/+^;hsp70l:Gq-Q209L* embryos (Fig. 7E,E′). Changes were also observed for *nr2f5*, an essential upper jaw specification factor (Barske et al., 2018) (Fig. 7C-D′,G-H′). In *edn1^+/+^* embryos, *nr2f5* expression was confined to the dorsal and intermediate domains of the first and second arches (Fig. 7C,C′), whereas expression expanded into the ventral domains of the first and second arches in *edn1^−/−^* embryos (Fig. 7D,D′). In *edn1^+/+^;hsp70l:Gq-Q209L* and *edn1^−/−^;hsp70l:Gq-Q209L* embryos, however, *nr2f5* was reduced overall (Fig. 7G-H′). These data indicate that Gq is sufficient for induction of lower jaw patterning genes and subsequent development of lower jaw structures.

## DISCUSSION

In this study, we addressed a long-standing question regarding the role of the Gq/11 family and potentially other Gα family members in lower jaw patterning (Sato et al., 2008a). Using the zebrafish model, we specifically interrogated the role of Gq/11 with three orthogonal approaches. We showed that inhibition of Gq/11 activity with a small molecule inhibitor or mutant alleles of *gna11a* and *gna11b* produced craniofacial phenotypes similar to those observed in *edn1^−/−^* larvae and larvae injected with morpholinos targeting *ednraa* and *ednrab* (Miller et al., 2000; Nair et al., 2007). We also showed that transgene-mediated expression of a constitutively active Gq protein, Gq-Q209L, can rescue lower jaw structures in *edn1^−/−^* embryos and can also transform the pterygoid process of the palatoquadrate to a Meckel's cartilage-like structure. Together, these results suggest that Gq/11signaling activity is necessary and sufficient to activate gene expression programs in cranial NCCs that establish lower jaw identity in zebrafish.

Our genetic analysis with mutant alleles has also revealed differential requirements for Gq/11 genes in zebrafish craniofacial development. Of the four members of the Gq/11 gene family – Gq, G11, G14 and G15 – we generated mutant alleles for Gq (*gnaq*) and G11 (*gna11a* and *gna11b*) only. Larvae that were homozygous mutants for both G11 genes (*gnaq^+/+^;gna11a^−/−^;gna11b^−/−^*) recapitulated the *edn1^−/−^* mutant phenotype (Figs 5C and 2G), suggesting that G11 genes are required for lower jaw patterning, whereas the Gq, G14 and G15 genes are not. The dispensability of G14 and G15 in lower jaw patterning is consistent with studies that have reported that expression of G14 and G15 genes is restricted to sensory organs and hematopoietic lineages (Ohmoto et al., 2011; Oka and Korsching, 2011). The dispensability of Gq was surprising, however, given its role in mouse lower jaw development (discussed further below). One of the most surprising findings from this work was the phenotypic discrepancy between zebrafish and mice lacking Gq and G11. The phenotype of *gna11a^−/−^;gna11b^−/−^* mutant zebrafish larvae are identical to *edn1* mutants (Miller et al., 2000), with both exhibiting severe hypoplasia of the Meckel's cartilage, fusion of the jaw joint and loss of the symplectic and ceratohyal cartilages. In contrast, craniofacial phenotypes in mice do not overlap between Edn1/Ednra pathway mutants and Gq/11 mutants. In embryonic day (E) 18.5 *Edn1^−/−^*, *Ednra^−/−^* and *Ece1^−/−^* mouse embryos, the entire mandible undergoes a homeotic transformation into a maxilla-like structure (Kurihara et al., 1995; Clouthier et al., 1998; Yanagisawa et al., 1998b). However, in E18.5 *Gnaq^flox/flox^;Gna11^−/−^;P0-Cre* mouse embryos, lower jaw defects were limited to the proximal two-thirds of the mandible (Dettlaff-Swiercz et al., 2005) and more closely resembled zebrafish embryos treated with YM (Figs 2 and 3) or reduced Gq/11 gene dosage (Fig. 5) than *Edn1^−/−^* or *Ednra^−/−^* mouse embryos (Clouthier et al., 1998; Ozeki et al., 2004). One explanation for the partial lower jaw defect in *Gnaq^flox/flox^;Gna11^−/−^;P0-Cre* embryos is that gene recombination in first pharyngeal arch NCCs induced by P0-Cre is less efficient compared to the NCC deletion strain *Wnt1-Cre* (Chen et al., 2017). Therefore, the residual levels of Gq expression and activity in cranial NCCs of *Gnaq^flox/flox^;Gna11^−/−^;P0-Cre* embryos may be sufficient to pattern the ventral domain but not the intermediate domain. This is consistent with numerous studies, including this one, that have reported that cranial NCCs in the intermediate domain are highly sensitive to perturbations that diminish Ednra signaling activity, such as treatment with YM (Figs 2 and 3) or an Ednra antagonist (Ruest and Clouthier, 2009), reduction of Gq/11 gene dosage (Fig. 5) (Offermanns et al., 1998), or reduction of Edn1 levels with an Edn1 morpholino (Miller and Kimmel, 2001) or a mutant allele of *furina* (Walker et al., 2006), an enzyme in the Edn1 biosynthetic pathway. This sensitivity of cranial NCCs in the intermediate domain has been attributed to a morphogen gradient model for Edn1, which posits that extracellular concentrations of Edn1 are highest near the source, the ventral ectoderm, and diminishes towards the dorsal regions of the arch (Kimmel et al., 2003). This question regarding the role of Gq/11 in mouse lower jaw development could be resolved with *Wnt1-Cre*, which has been shown to induce sufficient levels of gene recombination in cranial NCCs in *Ednra^flox/flox^* embryos (*Ednra^flox/flox^;Wnt1-Cre*) to recapitulate the *Ednra^−/−^* phenotype (Ruest and Clouthier, 2009).

The zebrafish mutant alleles for Gq/11 genes revealed two additional differences between zebrafish and mice. First, mice and zebrafish embryos have different genetic requirements for Gq and G11 genes. In mice with conventional Gq/11 knockout alleles, embryos with one wild-type allele for *Gna11* (*Gnaq^−/−^;Gna11^+/−^*) exhibited lower jaw defects, whereas embryos with one wild-type allele for *Gnaq* (*Gnaq^+/−^;Gna11^−/−^*) did not, indicating that *Gnaq* is primarily responsible for driving lower jaw patterning (Offermanns et al., 1998)*.* In zebrafish, *gna11a* and *gna11b* were required for lower jaw patterning, but *gnaq* was dispensable (Fig. 5). One possible explanation for this discrepancy is that *gnaq* does in fact contribute to lower jaw patterning in zebrafish, albeit to a lesser extent, with the loss of *gnaq* resulting in skeletal defects that are imperceptible with our current analysis. An alternative explanation is that *gnaq* is simply not expressed in the lower jaw progenitor cells and therefore does not contribute to lower jaw patterning in zebrafish. The latter explanation is consistent with our gene expression analyses using scRNA-seq (Fig. S6), qPCR (Fig. S7) and *in situ* hybridization (Fig. S8), which suggest that *gnaq* expression is absent or expressed at lower levels relative to *gna11a* and *gna11b* in cranial NCCs. This difference may reflect divergent regulation of Gq/11 genes between teleosts and mammals. The teleost genome, which has undergone an additional round of duplication compared with mammals, exhibits significant divergence in the cis-regulatory elements (CREs) of duplicated genes and paralogs that result in different spatial and temporal expression patterns compared with other vertebrate species (Kassahn et al., 2009). The CREs for Gq/11 genes in teleosts may have also undergone changes that result in expression patterns that differ from mammals. A comparative analysis of the CREs and expression profiles for Gq/11 genes in zebrafish and mice may provide insights into evolutionary mechanisms that drive changes to gene regulatory networks for cell signaling proteins, which could have implications for understanding how the form and function of anatomical structures have diverged between teleosts and mammals (Pires-daSilva and Sommer, 2003; de Mendoza et al., 2014).

The second difference was that zebrafish and mice are differentially sensitive to loss of Gq/11 signaling. *Gnaq^−/−^;Gna11^−/−^* mouse embryos exhibit mid-gestational lethality due, in part, to myocardial hypoplasia (Offermanns et al., 1998). However, zebrafish embryos that are triple homozygous for Gq/11 knockout alleles did not exhibit evidence of embryonic lethality prior to 6 dpf based on general morphology and normal Mendelian inheritance of alleles (Table S3). Although heart development was not examined in this study, it is possible that our triple homozygous Gq/11 mutant zebrafish have similar heart defects as mice given the conservation of Gq/11 function in craniofacial development. Unlike mouse embryos, zebrafish larvae can tolerate defective heart function up to 7 dpf due to a lack of dependence on oxygen from the circulatory system (Stainier et al., 1996). Therefore, this property of zebrafish physiology could be leveraged to examine the role of Gq/11 in heart development, which is not fully understood (Malbon, 2005; Wettschureck and Offermanns, 2005).

Our study also provides additional evidence for the utility and specificity of YM as a pharmacological tool to interrogate the role of Gq/11 *in vivo* (Shibata et al., 2022). Embryos treated with 100 μM YM (the highest concentration that we could use; see Materials and Methods) produced phenotype and gene expression changes similar to embryos with partial loss of Edn1/Ednra signaling (Figs 2 and 3) (Miller and Kimmel, 2001; Walker et al., 2006; Nair et al., 2007; Ruest and Clouthier, 2009). The window of sensitivity to YM also agrees with the window of sufficiency for endothelin signaling in lower jaw patterning from previous studies (Miller et al., 2000, 2003; Walker et al., 2006; Kimmel et al., 2007; Ruest and Clouthier, 2009; Vieux-Rochas et al., 2010; Alexander et al., 2011; Zuniga et al., 2011; Barske et al., 2016, 2018; Meinecke et al., 2018). Further, applying YM on *edn1* heterozygous mutants produced craniofacial phenotypes that were more severe in the spectrum of Edn1/Ednra-class phenotypes (Fig. 4). Although we acknowledge that the use of YM in zebrafish studies is limited by the concentration we can use, this additive effect of YM on sensitized backgrounds could be advantageous for studying other Gq/11-coupled receptors that might otherwise be embryonic lethal when knocked out. The effect of YM on cranial NCCs in zebrafish embryos is in stark contrast to the potency and efficacy of YM and its sister compound, FR900359, in cell culture assays, which exhibit an IC50 in the nanomolar concentration range (Nishimura et al., 2010; Schrage et al., 2015). The diminished inhibitory effect of YM in zebrafish embryos may be caused by poor perfusion into relevant tissues, inefficient cell permeability, or degradation. FR900359 exhibits slightly different physicochemical properties (Schlegel et al., 2021) and thus may exhibit greater effects on zebrafish embryos than YM.

By establishing Gq/11 as the sole signaling mediator for Ednra in lower jaw patterning, we can begin to elucidate mechanisms of jaw patterning that remain poorly characterized. As demonstrated by our study and others, correct positioning of the upper and lower jaw patterning domains requires a specific balance of signaling inputs by Edn1/Ednra, Jagged/Notch and Nr2f nuclear receptors (Sato et al., 2008b; Zuniga et al., 2010; Alexander et al., 2011; Tavares and Clouthier, 2015; Barske et al., 2016, 2018; Sucharov et al., 2019; Kurihara et al., 2023). It remains unknown how these signaling pathways are integrated to elicit specific transcriptional outputs that refine the positional identities of cranial NCCs residing in and around the upper jaw–lower jaw boundary. Future studies will examine mechanisms of signal integration.

## MATERIALS AND METHODS

### Protein sequence alignment

Protein sequences were aligned using Clustal Omega (Madeira et al., 2019). The following are accession numbers for sequences used. For human proteins: Gq (NP_002063.2), G11 (NP_002058.2) and Gi1 (NP_002060.4). For zebrafish proteins: Gq (NP_001138271.1), G11a (NP_001038501.1), G11b (NP_001007774.1), G14 (NP_001003753.1), G14a (XP_683989.2), G15.1 (NP_001003626.2), G15.2 (XP_002667410.2), G15.3 (translated from mRNA sequence for *gna15.3*, XR_659583.3), G15.4 (NP_001038454.1), Gi1 (NP_957265.1), Gs (XP_001335732.1) and G12a (NP_001013295.1).

### Zebrafish strains and husbandry

All work was approved by the University of Colorado Institutional Animal Care and Use Committee (Protocol No. 00188). Zebrafish (*Danio rerio*) adults and embryos were raised and staged according to Kimmel et al. (1995) and Westerfield (2007). Mutant and transgenic lines were maintained in the AB strain. The following mutant and transgenic lines were previously described: *sucker/edn1^tf216^* (Miller et al., 2000), *Tg(fli1a:EGFP)^γ1^* (Lawson and Weinstein, 2002) and *Tg(sox10:mRFP)^vu234^* (Kirby et al., 2006). In addition, five new lines were created: mutant alleles for *edn1^co3009^*, *gnaq^co3010^*, *gna11a^co3015^* and *gna11b^co3014^*, and the transgenic line *Tg(hsp70l:Gq-Q209L-IRES-EGFP,cmlc2:EGFP).* All lines are available upon request. Construction of strains is described below.

#### Generation of deletion alleles

All mutant alleles were generated with the CRISPR/Cas9 gene-editing system as previously described (Hwang et al., 2013; Jao et al., 2013). Gene-specific target sequences were generated with CHOPCHOP v.2 (Labun et al., 2016), and guide RNAs (gRNAs) were generated as previously described (Bassett et al., 2013). To generate *edn1^co3009^*, two target sequences were identified in exon 2. To generate *gnaq^co3010^* and *gna11b^co3014^*, target sequences were identified in exon 4 (Fig. S9). To generate *gna11a^co3015^*, a target sequence was identified in exon 3 (Fig. S9). The following are the target sequences used for each gene: *edn1*, 5′-GGAATAAGAGATGCTCCTGC-3′and 5′-GGACATAATATGGGTGAACA-3′; *gnaq*, 5′-GGCTGGGTGGGAATGTAGGAA-3′; *gna11a*, 5′-GGCGAGAGGTCGATGTCGAGA-3′; *gna11b*, 5′GGTGGGAAGGTACGAAGATT-3′. The individual target sequences were then incorporated into an oligo consisting of 5′-[T7 promoter sequence]-[Target sequence]-[Start of scaffold sequence]-3′. The T7 promoter sequence is 5′-AATTAATACGACTCACTATA-3′, and the start of the scaffold sequence is 5′-GTTTTAGAGCTAGAAATAGC-3′. DNA templates for gene-specific gRNAs were then generated by performing PCR with the target sequence-containing oligo and a separate oligo containing the gRNA scaffold sequence: 5′-GATCCGCACCGACTCGGTGCCACTTTTTCAAGTTGATAACGGACTAGCCTTATTTTAACTTGCTATTTCTAGCTCTAAAAC-3′. All oligos were purchased from IDT DNA Technologies. PCR reactions were performed with KAPA HiFi HotStart ReadyMix (Roche, 07958927001) (1× KAPA Taq, 500 nM target sequence oligo, 500 nM gRNA scaffold oligo) using the following cycling parameters: 98°C for 30 s; 40 cycles of 98°C for 10 s, 60°C for 10 s; 72°C for 15 s; 72°C for 10 min. Following purification of the PCR product using the QIAquick PCR Purification Kit (QIAGEN, 28104), the gRNA was synthesized using the MEGAscript T7 Transcription kit (Invitrogen, AM1334). Cas9 mRNA (codon-optimized for zebrafish) was generated as previously described (Jao et al., 2013). Briefly, pT3TS-nCas9n (Addgene plasmid #46757) was linearized with XbaI restriction enzyme and then used as the template for *in vitro* transcription using the mMESSAGE mMACHINE T3 Transcription Kit (Invitrogen, AM1348).

Embryos were injected at the one-cell stage with 2 nl of solution containing 100 ng/µl Cas9 mRNA, 50 ng/µl gRNA and 0.025% Phenol Red (Sigma-Aldrich, P0290). To determine whether the gRNAs were excising the genomic loci of interest, at least ten embryos per injected clutch were lysed in genomic DNA extraction buffer (10 mM Tris pH 8, 2 mM EDTA, 0.2% Triton X-100, 0.2 mg/ml Proteinase K) at 24 hpf, and the genomic loci of interest were PCR-amplified with genotyping primers. PCR amplicons were then analyzed by gel electrophoresis, with the presence of smears or ladders serving as indicators of potential genomic lesions. Clutches indicating genomic lesions were grown to adults (F0) and then crossed with wild-type ABs to generate F1 embryos. F1 adults were screened for genomic lesions at loci of interest by PCR, and the nature of the genomic lesions were characterized by Sanger sequencing. F1 adults harboring frameshift mutations were crossed with wild-type ABs to identify those that transmit mutant alleles to offspring in the expected Mendelian frequency. Germ line-stable F1 adults were kept and outcrossed to wild-type ABs to generate F2 fish used in this study. The following genotyping primers were used for each allele: *edn1^co3009^*, 5′-TAGGTGCTCCAGCATCTTTG-3′ and 5′-GGAGCGTTTCCAAGTCCATA-3′; *gnaq^co3010^*, 5′-TATGATGCCTTTTTGTCCACAG-3′ and 5′-CATTGTCAGACTCGACGAGAA-3′; *gna11a^co3015^*, 5′-TCAAGTCGTAAAATGGGTTGTG-3′ and 5′-TAAAAGCAGCAAATGACGACAC-3′; *gna11b^co3014^*, 5′-GAAGCATCCTTTACCAAACCAC-3′ and 5′-CGAACGAAGGCAGATGAATAAT-3′. The frameshift mutation in the *gna11b^co3014^* allele introduces a BceAI restriction site. Thus, PCR reactions from the *gna11b^co3014^* genotyping assay were digested with BceAI to further distinguish PCR amplicons from wild-type and mutant alleles.

#### Generation of transgenic line

The *hsp70l:Gq-Q209L-IRES-EGFP,cmlc2:EGFP* plasmid was generated using the Tol2kit as described by Kwan et al. (2007). First, the middle entry vector containing Gq-Q209L, pME-Gq-Q209L, was generated. The Gq-Q209L cDNA was PCR-amplified from pc3.1-Gq-Q209L (Kanai et al., 2022) and then cloned into the pME-MCS vector using T4 ligase (New England Biolabs, M0202). pME-Gq-Q209L was then combined with p5E-hsp70l, p3E-IRES-GFPpA and pDestTol2CG2 using LR Clonase II Plus Enzyme Mix (Invitrogen,12538-120). To generate transposase mRNA, the pCS2FA-transposase plasmid was linearized with NotI restriction enzyme and then used as the template for *in vitro* transcription using the mMESSAGE mMACHINE SP6 Transcription Kit (Invitrogen, AM1340).

Embryos at the one-cell stage were injected with a 2 nl solution containing 12.5 ng/µl transposase mRNA, 12.5 ng/µl *hsp70l:Gq-Q209L-IRES-EGFP,cmlc2:EGFP* and 0.025% Phenol Red*.* Injected embryos were grown to adults (F0) and then crossed to wild-type ABs. Offspring (F1) were screened for the presence of the transgene by examining GFP expression in the heart. Transgene-positive, germ line-stable F1 fish were then crossed into *edn1^+/−^* fish to generate *Tg(hsp70l:GqQ209L-IRES-EGFP); edn1^+/−^* fish. The plasmids pME-MCS, p5E-hsp70l, p3E-IRES-GFPpA, pDestTol2CG2 and pCS2FA-transposase were provided by Kristen Kwan (Kwan et al., 2007).

### YM incubation

Embryos generated by intercrossing wild-type ABs were dechorionated at 14 hpf in Petri dishes coated with 0.5% agarose using E2 media (Westerfield, 2007) containing 2 mg/ml Pronase (Roche, 10165921001) at 25°C for 10-15 min. Embryos were subsequently washed in fresh E2 media in agarose-coated Petri dishes three times. Dechorionated embryos in agarose-coated plates were then incubated with DMSO or YM [Adipogen (AG-CN2-0509-M001) or Cayman Chemicals (29735)] dissolved in DMSO, from 16 to 36 hpf. At 36 hpf, embryos were washed with fresh E2 media three times and then processed for skeletal preparations at 6 dpf. Embryos for fluorescence *in situ* hybridization with immunofluorescence were fixed overnight at 36 hpf in 4% paraformaldehyde (PFA) in PBS. Note that the product datasheet for YM states that the solubility limit in DMSO is 10 mM (9.6 mg/ml), which is the stock concentration we used. However, for zebrafish embryo media, 1% DMSO is the upper limit that does not cause developmental defects (Hoyberghs et al., 2021). To ensure that we did not exceed 1% DMSO in the embryo media, 100 µM YM was the highest working concentration we could use.

### Skeletal preparations

Larvae were stained with Alizarin Red and Alcian Blue as previously described (Walker and Kimmel, 2007; Brooks and Nichols, 2017).

### scRNA-seq

#### Sample preparation for scRNA-seq

Cranial neural crest cells were isolated from zebrafish embryos using a *fli1a:EGFP* and *sox10:mRFP* double labeling technique as previously described (Barske et al., 2016; Askary et al., 2017; Mitchell et al., 2021) with some modifications. In brief, 140 double-positive embryos were dechorionated at 14 hpf with 2 mg/ml Pronase. Seventy embryos were then treated with 100 µM YM or DMSO from 16 to 36 hpf. At 36 hpf, ten embryos from each condition were set aside for skeletal preparations at 6 dpf. The heads of the remaining embryos were severed, pooled and de-yolked by gentle pipetting in Ca^2+^-free Ringer's solution (116 mM NaCl, 2.9 mM KCl, 5 mM HEPES, pH 7.0). The heads were then dissociated into single cells with mechanical agitation and enzymatic digestion for 15 min at 4°C with cold-activated protease from *Bacillus licheniformis* (Sigma-Aldrich, P5380) in dissociation buffer (10 mg/ml *B. licheniformis* protease, 125 U/ml DNAse, 2.5 mM EDTA, PBS), followed by enzyme neutralization with stop solution (30% fetal bovine serum, 0.8 mM CaCl_2_, in PBS). Cells were then centrifuged (400 ***g*** for 5 min), resuspended in cell suspension buffer [1% fetal bovine serum, 0.8 mM CaCl_2,_ Leibovitz's L-15 Medium (Gibco, 21083-027)], filtered through a 70 µm strainer (PluriSelect, 43-10040-40), centrifuged again (400 ***g*** for 5 min) and resuspended in sorting buffer (1% fetal bovine serum, 1 mM EDTA, 25 mM HEPES). EGFP and mRFP double-positive cells were then purified by FACS using a MoFlo XDP100 (Beckman Coulter). A total of 72,000 and 86,000 double-positive cells were purified for DMSO and YM-treated samples, respectively. Cells were once again centrifuged (400 ***g*** for 5 min) and resuspended in cell suspension buffer. After confirming cell viability with Trypan Blue (Gibco, 15250-061), 8000 cells for each condition were submitted to the University of Colorado Anschutz Medical Campus Genomics and Microarray Core for scRNA-seq. Single-cell capture, barcoding and library generation was performed using 10x Genomics single cell RNA-sequencing technology (Chromium Controller and Next GEM single Cell 3′ Kit v.3.1, Dual Index). Libraries were processed for paired-end sequencing with a read depth of 75,000/cell using NovaSeq6000 (Illumina).

#### scRNAseq analysis

Sequence reads were aligned to the *D. rerio* reference genome (GRCz11) and converted to count matrices with Cell Ranger v.5.0.1 (10x Genomics). Subsequent analyses of count matrices were performed with Seurat v.4.3.0 in R studio (Hao et al., 2021). Individual Seurat objects were created for DMSO and YM-treated samples, and cells in each dataset were filtered for quality. For DMSO-treated samples, 3700 cells were obtained after filtering for cells with 200-3000 unique feature counts and less than 5% mitochondrial DNA. For YM-treated cells, 4506 cells were obtained after filtering for cells with 200-3500 unique feature counts and less than 5% mitochondrial DNA. Datasets for DMSO or YM-treated samples were then merged, normalized and scaled, with cell cycle phase-associated genes regressed out. A list of S- and G2M-phase genes built into Seurat was converted to zebrafish identifiers using BioMart and then used for cell cycle regression (Nestorowa et al., 2016). Converted gene lists were manually checked and edited for any changes in nomenclature/duplications between the reference and Ensembl database. Linear dimensional reduction was then performed with principal component analysis (PCA), and the top 30 PCAs were used to generate and visualize clusters with the uniform manifold approximation and projection (UMAP) nonlinear dimensional reduction technique. Marker genes for clusters were obtained with a Wilcoxon Rank Sum test, and cluster identities were determined using published, experimentally verified marker genes (Nair et al., 2007; Talbot et al., 2010; Askary et al., 2017; Barske et al., 2018, 2016; Fabian et al., 2022). The approximate population of cells representing the intermediate domain were determined with a feature plot encompassing known intermediate domain marker genes, *ccn2b*, *dlx3b*, *dlx4a*, *dlx4b*, *emx2*, *fgfbp2a*, *foxc1b*, *foxd1*, *fsta*, *grem2b*, *igfbp5b*, *msx1a*, *nkx3-2* and *shox* (Fig. S4). Differential expression analysis was performed using Wilcoxon Rank Sum test between equivalent clusters of cells in DMSO and YM-treated samples.

### Quantitative PCR

RNA was collected from 40 heads of 28 hpf zebrafish embryos using Direct-zol RNA MiniPrep Kit (Zymo Research, R2050) following the manufacturer's protocol. cDNA was generated using the QuantiTect Reverse Transcription Kit (QIAGEN, 205311). qPCR assays were prepared using the QuantiTect SYBR Green PCR kit (QIAGEN, 204143) following the manufacturer's protocol and read in a CFX Connect Real-Time PCR Detection System (Bio-Rad). The following QuantiTect Primers were purchased from QIAGEN (249900): *gnaq* (GeneGlobe ID: QT02091502), *gna11a* (GeneGlobe ID: QT02065042), *gna11b* (GeneGlobe ID: QT02062060), *actb1* (GeneGlobe ID: QT02174907). The primer pair for *dlx2a* was: 5′-gaaacgctttcggccccta-3′ and 5′-ccattcggatttcaggttcgc-3′ (Weinschutz Mendes et al., 2020). *actb1* was used as the reference gene to calculate relative gene expression.

### *In situ* hybridization and immunofluorescence

Colorimetric *in situ* hybridization was performed as previously described (Thisse and Thisse, 2008). Two-color fluorescence *in situ* hybridization was performed as previously described (Talbot et al., 2010). Fluorescence *in situ* hybridization with immunofluorescence was performed as previously described (Mitchell et al., 2021). The following probes have been previously described: *dlx2a* (Akimenko et al., 1994), *dlx4b* (Ellies et al., 1997), *dlx5a* (Walker et al., 2006) and *nr2f5* (Barske et al., 2018)*.* The plasmid for *nr2f5* was provided by Lindsey Barske (Cincinnati Children's Hospital Medical Center, OH, USA). To generate probes for *gnaq*, *gna11a* and *gna11b*, gene fragments were first cloned into the pCRII plasmid with the TOPO-TA Cloning Kit (Invitrogen, 450640) following the manufacturer's protocol. The following primer pairs were used to amplify gene fragments: *gnaq*, 5′-cgttaacacgggaggaacac-3′ and 5′-gagctgtcggtcgatctcat-3′ (Takeuchi et al., 2017); *gna11a*, 5′-tactcgcacttcacctgtgc-3′ and 5′-tgggaaaggcgttttatttg-3′; and *gna11b*, 5′-caccgacacagagaacatcc-3′ and 5′-acattcatcgatgcgagttg-3′.

### Microscopy

Embryos processed for whole-mount fluorescence *in situ* hybridization and immunofluorescence were mounted in 0.4% agarose in PBS on a glass-bottom dish (MatTek, P35G-1.5-10-C), with images taken using a 20× air objective on a DMi8 microscope (Leica) equipped with an Andor Dragonfly confocal unit (Oxford Instruments). Images were subsequently processed using Imaris image analysis software (Oxford Instruments). Embryos processed for whole-mount colorimetric *in situ* hybridization and skeletal preparations were imaged on a SZX12 stereo microscope (Olympus) equipped with a SC100 camera (Olympus). Sections for colorimetric *in situ* hybridization were imaged using a 20× objective on a BX51 microscope (Olympus) under Nomarski optics, with images captured using a DP71 camera (Olympus). Sections processed for immunohistochemistry were imaged with a 63× oil immersion objective on a SP8 confocal microscope (Leica), and image processing was subsequently performed in Fiji (Schindelin et al., 2012).

### Heat shock protocol

Embryos generated from crossing *Tg(hsp70l:GqQ209L-IRES-GFP,cmlc2:EGFP);edn1^+/−^* and *edn1^+/−^* adults were heat-shocked at 16 hpf for 10 min in a 38°C water bath and then returned to 28.5°C. Transgenic and non-transgenic embryos were separated based on cardiac EGFP expression and monitored daily until 4 dpf to account for embryos developing cardiac edema, and then processed for skeletal preparations. Embryos processed for *in situ* hybridization were fixed overnight in 4% PFA in PBS at 28 hpf.

### Immunohistochemistry

Zebrafish embryos collected from *hsp70l:Gq-Q209L* and *sox10:mRFP* mating pairs were heat-shocked at 21 hpf and fixed 3 h later at 24 hpf in 4% PFA overnight at 4°C. Embryos were washed in PBS for 5 min three times, successively incubated in 15% sucrose/PBS and 30% sucrose/PBS, embedded in Tissue-Tek O.C.T. Compound (Sakura, 4583), and flash-frozen in an ethanol/dry ice bath. Embryos were then sectioned at 20 μm on a CM1900 cryostat (Leica). Sections were hydrated in PBS for 5 min, permeabilized in 0.1% Triton X-100/PBS (PBS-Triton) for 5 min, and then incubated in blocking buffer (2% bovine serum albumin, 2% goat serum, 0.1% Triton X-100, PBS) for 2 h at room temperature. Sections were then incubated in blocking buffer containing 1:200 dilution of primary antibody [rabbit P-p44/42MAPK (T202/Y204), Cell Signaling Technology, 4370, lot #17)] overnight at 4°C. Sections were washed for 15 min in PBS-Triton three times and then incubated in blocking buffer containing 1:500 dilution of secondary antibody (goat anti-rabbit Alexa 647, Invitrogen, A21245) for 2 h at room temperature. Sections were then washed in PBS-Triton for 15 min×3, incubated in PBS containing 0.1 µg/ml Hoechst 33342 (Invitrogen, H1399) for 15 min, washed in PBS for 5 min, and mounted in Prolong Gold Antifade Reagent (Invitrogen, P36930).

### Statistical analysis

Statistical analyses for the one-way ANOVA with Holm–Šídák multiple comparisons test, chi-square tests and unpaired *t*-tests were performed in Prism (GraphPad).

## Supplementary Material



10.1242/develop.204396_sup1 Supplementary information

Table S1. Marker genes for clusters generated from the integrated dataset comprising DMSO- and YM-treated samples. The numbers in the “cluster” column correspond to the numbers in the UMAPs presented below. These UMAPs are identical to Figure 3B, though with clusters labeled with numbers rather than names of specific NCC populations. The names of the cranial NCC populations are indicated in the adjacent column labeled “cell population”.

Table S2. Differentially expressed genes in DMSO- versus YM-treated samples. Differential expression analysis was performed between equivalent clusters in DMSO- and YM-treated samples. Cluster identities are indicated by the numbers and names in the “cluster” and “cell population” columns, respectively, which correspond to the UMAPs shown below. These UMAPs are identical to those in Figure 3B, with clusters labeled with numbers rather than names of specific NCC populations. The “avg_log2FC” values indicate differential expression in DMSO-treated samples relative to YM-treated samples.

## References

[DEV204396C1] Akimenko, M. A., Ekker, M., Wegner, J., Lin, W. and Westerfield, M. (1994). Combinatorial expression of three zebrafish genes related to distal-less: part of a homeobox gene code for the head. *J. Neurosci.* 14, 3475-3486. 10.1523/JNEUROSCI.14-06-03475.1994 7911517 PMC6576961

[DEV204396C2] Alexander, C., Zuniga, E., Blitz, I. L., Wada, N., Le Pabic, P., Javidan, Y., Zhang, T., Cho, K. W., Crump, J. G. and Schilling, T. F. (2011). Combinatorial roles for BMPs and Endothelin 1 in patterning the dorsal-ventral axis of the craniofacial skeleton. *Development* 138, 5135-5146. 10.1242/dev.067801 22031543 PMC3210495

[DEV204396C3] Annala, S., Feng, X., Shridhar, N., Eryilmaz, F., Patt, J., Yang, J., Pfeil, E. M., Cervantes-Villagrana, R. D., Inoue, A., Haberlein, F. et al. (2019). Direct targeting of Galpha(q) and Galpha(11) oncoproteins in cancer cells. *Sci. Signal.* 12, eaau5948. 10.1126/scisignal.aau5948 30890659

[DEV204396C4] Aramori, I. and Nakanishi, S. (1992). Coupling of two endothelin receptor subtypes to differing signal transduction in transfected Chinese hamster ovary cells. *J. Biol. Chem.* 267, 12468-12474. 10.1016/S0021-9258(18)42300-9 1319997

[DEV204396C5] Askary, A., Xu, P., Barske, L., Bay, M., Bump, P., Balczerski, B., Bonaguidi, M. A. and Crump, J. G. (2017). Genome-wide analysis of facial skeletal regionalization in zebrafish. *Development* 144, 2994-3005. 10.1242/dev.151712 28705894 PMC5592815

[DEV204396C6] Barske, L., Askary, A., Zuniga, E., Balczerski, B., Bump, P., Nichols, J. T. and Crump, J. G. (2016). Competition between jagged-notch and endothelin1 signaling selectively restricts cartilage formation in the zebrafish upper face. *PLoS Genet.* 12, e1005967. 10.1371/journal.pgen.1005967 27058748 PMC4825933

[DEV204396C7] Barske, L., Rataud, P., Behizad, K., Del Rio, L., Cox, S. G. and Crump, J. G. (2018). Essential role of Nr2f nuclear receptors in patterning the vertebrate upper jaw. *Dev. Cell* 44, 337-347.e5. 10.1016/j.devcel.2017.12.022 29358039 PMC5801120

[DEV204396C8] Bassett, A. R., Tibbit, C., Ponting, C. P. and Liu, J. L. (2013). Highly efficient targeted mutagenesis of Drosophila with the CRISPR/Cas9 system. *Cell Rep* 4, 220-228. 10.1016/j.celrep.2013.06.020 23827738 PMC3714591

[DEV204396C9] Brooks, E. P. and Nichols, J. T. (2017). Shifting zebrafish lethal skeletal mutant penetrance by progeny testing. *J. Vis. Exp.* 127, 56200. 10.3791/56200 PMC5614404 28892034

[DEV204396C10] Chai, Y., Jiang, X., Ito, Y., Bringas, P., Jr, Han, J., Rowitch, D. H., Soriano, P., McMahon, A. P. and Sucov, H. M. (2000). Fate of the mammalian cranial neural crest during tooth and mandibular morphogenesis. *Development* 127, 1671-1679. 10.1242/dev.127.8.1671 10725243

[DEV204396C11] Chen, G., Ishan, M., Yang, J., Kishigami, S., Fukuda, T., Scott, G., Ray, M. K., Sun, C., Chen, S. Y., Komatsu, Y. et al. (2017). Specific and spatial labeling of P0-Cre versus Wnt1-Cre in cranial neural crest in early mouse embryos. *Genesis* 55, dvg.23034. 10.1002/dvg.23034 PMC5473950 28371069

[DEV204396C12] Clouthier, D. E., Hosoda, K., Richardson, J. A., Williams, S. C., Yanagisawa, H., Kuwaki, T., Kumada, M., Hammer, R. E. and Yanagisawa, M. (1998). Cranial and cardiac neural crest defects in endothelin-A receptor-deficient mice. *Development* 125, 813-824. 10.1242/dev.125.5.813 9449664

[DEV204396C13] Clouthier, D. E., Williams, S. C., Yanagisawa, H., Wieduwilt, M., Richardson, J. A. and Yanagisawa, M. (2000). Signaling pathways crucial for craniofacial development revealed by endothelin-A receptor-deficient mice. *Dev. Biol.* 217, 10-24. 10.1006/dbio.1999.9527 10625532

[DEV204396C14] de Mendoza, A., Sebe-Pedros, A. and Ruiz-Trillo, I. (2014). The evolution of the GPCR signaling system in eukaryotes: modularity, conservation, and the transition to metazoan multicellularity. *Genome Biol. Evol.* 6, 606-619. 10.1093/gbe/evu038 24567306 PMC3971589

[DEV204396C15] Dettlaff-Swiercz, D. A., Wettschureck, N., Moers, A., Huber, K. and Offermanns, S. (2005). Characteristic defects in neural crest cell-specific Galphaq/Galpha11- and Galpha12/Galpha13-deficient mice. *Dev. Biol.* 282, 174-182. 10.1016/j.ydbio.2005.03.006 15936338

[DEV204396C16] Ellies, D. L., Stock, D. W., Hatch, G., Giroux, G., Weiss, K. M. and Ekker, M. (1997). Relationship between the genomic organization and the overlapping embryonic expression patterns of the zebrafish dlx genes. *Genomics* 45, 580-590. 10.1006/geno.1997.4978 9367683

[DEV204396C17] Fabian, P., Tseng, K. C., Thiruppathy, M., Arata, C., Chen, H. J., Smeeton, J., Nelson, N. and Crump, J. G. (2022). Lifelong single-cell profiling of cranial neural crest diversification in zebrafish. *Nat. Commun.* 13, 13. 10.1038/s41467-021-27594-w 35013168 PMC8748784

[DEV204396C18] Flock, T., Ravarani, C. N. J., Sun, D., Venkatakrishnan, A. J., Kayikci, M., Tate, C. G., Veprintsev, D. B. and Babu, M. M. (2015). Universal allosteric mechanism for Galpha activation by GPCRs. *Nature* 524, 173-179. 10.1038/nature14663 26147082 PMC4866443

[DEV204396C19] Gilman, A. G. (1987). G proteins: transducers of receptor-generated signals. *Annu. Rev. Biochem.* 56, 615-649. 10.1146/annurev.bi.56.070187.003151 3113327

[DEV204396C20] Gordon, C. T., Petit, F., Kroisel, P. M., Jakobsen, L., Zechi-Ceide, R. M., Oufadem, M., Bole-Feysot, C., Pruvost, S., Masson, C., Tores, F. et al. (2013). Mutations in endothelin 1 cause recessive auriculocondylar syndrome and dominant isolated question-mark ears. *Am. J. Hum. Genet.* 93, 1118-1125. 10.1016/j.ajhg.2013.10.023 24268655 PMC3853412

[DEV204396C21] Gordon, C. T., Weaver, K. N., Zechi-Ceide, R. M., Madsen, E. C., Tavares, A. L., Oufadem, M., Kurihara, Y., Adameyko, I., Picard, A., Breton, S. et al. (2015). Mutations in the endothelin receptor type A cause mandibulofacial dysostosis with alopecia. *Am. J. Hum. Genet.* 96, 519-531. 10.1016/j.ajhg.2015.01.015 25772936 PMC4385188

[DEV204396C22] Halloran, M. C., Sato-Maeda, M., Warren, J. T., Su, F., Lele, Z., Krone, P. H., Kuwada, J. Y. and Shoji, W. (2000). Laser-induced gene expression in specific cells of transgenic zebrafish. *Development* 127, 1953-1960. 10.1242/dev.127.9.1953 10751183

[DEV204396C23] Hao, Y., Hao, S., Andersen-Nissen, E., Mauck, W. M., III, Zheng, S., Butler, A., Lee, M. J., Wilk, A. J., Darby, C., Zager, M. et al. (2021). Integrated analysis of multimodal single-cell data. *Cell* 184, 3573-3587.e29. 10.1016/j.cell.2021.04.048 34062119 PMC8238499

[DEV204396C24] Hoyberghs, J., Bars, C., Ayuso, M., Van Ginneken, C., Foubert, K. and Van Cruchten, S. (2021). DMSO concentrations up to 1% are safe to be used in the zebrafish embryo developmental toxicity assay. *Front. Toxicol.* 3, 804033. 10.3389/ftox.2021.804033 35295145 PMC8915880

[DEV204396C107] Huggins, J. P., Pelton, J. T. and Miller, R. C. (1993). The structure and specificity of endothelin receptors: their importance in physiology and medicine. *Pharmacol. Ther.* 59, 55-123. 10.1016/0163-7258(93)90041-b 8259382

[DEV204396C25] Hwang, W. Y., Fu, Y., Reyon, D., Maeder, M. L., Tsai, S. Q., Sander, J. D., Peterson, R. T., Yeh, J. R. and Joung, J. K. (2013). Efficient genome editing in zebrafish using a CRISPR-Cas system. *Nat. Biotechnol.* 31, 227-229. 10.1038/nbt.2501 23360964 PMC3686313

[DEV204396C26] Inoue, A., Raimondi, F., Kadji, F. M. N., Singh, G., Kishi, T., Uwamizu, A., Ono, Y., Shinjo, Y., Ishida, S., Arang, N. et al. (2019). Illuminating G-protein-coupling selectivity of GPCRs. *Cell* 177, 1933-1947.e25. 10.1016/j.cell.2019.04.044 31160049 PMC6773469

[DEV204396C27] Jao, L. E., Wente, S. R. and Chen, W. (2013). Efficient multiplex biallelic zebrafish genome editing using a CRISPR nuclease system. *Proc. Natl. Acad. Sci. USA* 110, 13904-13909. 10.1073/pnas.1308335110 23918387 PMC3752207

[DEV204396C28] Jeong, J., Li, X., McEvilly, R. J., Rosenfeld, M. G., Lufkin, T. and Rubenstein, J. L. (2008). Dlx genes pattern mammalian jaw primordium by regulating both lower jaw-specific and upper jaw-specific genetic programs. *Development* 135, 2905-2916. 10.1242/dev.019778 18697905 PMC4913551

[DEV204396C29] Jiang, M., Gold, M. S., Boulay, G., Spicher, K., Peyton, M., Brabet, P., Srinivasan, Y., Rudolph, U., Ellison, G. and Birnbaumer, L. (1998). Multiple neurological abnormalities in mice deficient in the G protein Go. *Proc. Natl. Acad. Sci. USA* 95, 3269-3274. 10.1073/pnas.95.6.3269 9501252 PMC19731

[DEV204396C30] Kalinec, G., Nazarali, A. J., Hermouet, S., Xu, N. and Gutkind, J. S. (1992). Mutated alpha subunit of the Gq protein induces malignant transformation in NIH 3T3 cells. *Mol. Cell. Biol.* 12, 4687-4693. 10.1128/mcb.12.10.4687-4693.1992 1328859 PMC360395

[DEV204396C31] Kanai, S. M., Heffner, C., Cox, T. C., Cunningham, M. L., Perez, F. A., Bauer, A. M., Reigan, P., Carter, C., Murray, S. A. and Clouthier, D. E. (2022). Auriculocondylar syndrome 2 results from the dominant-negative action of PLCB4 variants. *Dis. Model. Mech.* 15, dmm049320. 10.1242/dmm.049320 35284927 PMC9066496

[DEV204396C32] Kassahn, K. S., Dang, V. T., Wilkins, S. J., Perkins, A. C. and Ragan, M. A. (2009). Evolution of gene function and regulatory control after whole-genome duplication: comparative analyses in vertebrates. *Genome Res.* 19, 1404-1418. 10.1101/gr.086827.108 19439512 PMC2720184

[DEV204396C33] Kawanabe, Y., Okamoto, Y., Nozaki, K., Hashimoto, N., Miwa, S. and Masaki, T. (2002). Molecular mechanism for endothelin-1-induced stress-fiber formation: analysis of G proteins using a mutant endothelin(A) receptor. *Mol. Pharmacol.* 61, 277-284. 10.1124/mol.61.2.277 11809851

[DEV204396C34] Khac, L. D., Naze, S. and Harbon, S. (1994). Endothelin receptor type A signals both the accumulation of inositol phosphates and the inhibition of cyclic AMP generation in rat myometrium: stimulation and desensitization. *Mol. Pharmacol.* 46, 485-494. 10.1016/S0026-895X(25)09725-1 7935329

[DEV204396C35] Kimmel, C. B., Ballard, W. W., Kimmel, S. R., Ullmann, B. and Schilling, T. F. (1995). Stages of embryonic development of the zebrafish. *Dev. Dyn.* 203, 253-310. 10.1002/aja.1002030302 8589427

[DEV204396C36] Kimmel, C. B., Ullmann, B., Walker, M., Miller, C. T. and Crump, J. G. (2003). Endothelin 1-mediated regulation of pharyngeal bone development in zebrafish. *Development* 130, 1339-1351. 10.1242/dev.00338 12588850

[DEV204396C37] Kimmel, C. B., Walker, M. B. and Miller, C. T. (2007). Morphing the hyomandibular skeleton in development and evolution. *J. Exp. Zool. B Mol. Dev. Evol.* 308, 609-624. 10.1002/jez.b.21155 17358015

[DEV204396C38] Kirby, B. B., Takada, N., Latimer, A. J., Shin, J., Carney, T. J., Kelsh, R. N. and Appel, B. (2006). In vivo time-lapse imaging shows dynamic oligodendrocyte progenitor behavior during zebrafish development. *Nat. Neurosci.* 9, 1506-1511. 10.1038/nn1803 17099706

[DEV204396C39] Kleuss, C., Raw, A. S., Lee, E., Sprang, S. R. and Gilman, A. G. (1994). Mechanism of GTP hydrolysis by G-protein alpha subunits. *Proc. Natl. Acad. Sci. USA* 91, 9828-9831. 10.1073/pnas.91.21.9828 7937899 PMC44910

[DEV204396C40] Kurihara, Y., Kurihara, H., Oda, H., Maemura, K., Nagai, R., Ishikawa, T. and Yazaki, Y. (1995). Aortic arch malformations and ventricular septal defect in mice deficient in endothelin-1. *J. Clin. Invest.* 96, 293-300. 10.1172/JCI118033 7615798 PMC185200

[DEV204396C41] Kurihara, Y., Ekimoto, T., Gordon, C. T., Uchijima, Y., Sugiyama, R., Kitazawa, T., Iwase, A., Kotani, R., Asai, R., Pingault, V. et al. (2023). Mandibulofacial dysostosis with alopecia results from ETAR gain-of-function mutations via allosteric effects on ligand binding. *J. Clin. Invest.* 133, e151536. 10.1172/JCI151536 36637912 PMC9927936

[DEV204396C42] Kwan, K. M., Fujimoto, E., Grabher, C., Mangum, B. D., Hardy, M. E., Campbell, D. S., Parant, J. M., Yost, H. J., Kanki, J. P. and Chien, C. B. (2007). The Tol2kit: a multisite gateway-based construction kit for Tol2 transposon transgenesis constructs. *Dev. Dyn.* 236, 3088-3099. 10.1002/dvdy.21343 17937395

[DEV204396C43] Labun, K., Montague, T. G., Gagnon, J. A., Thyme, S. B. and Valen, E. (2016). CHOPCHOP v2: a web tool for the next generation of CRISPR genome engineering. *Nucleic Acids Res.* 44, W272-W276. 10.1093/nar/gkw398 27185894 PMC4987937

[DEV204396C44] Lapadula, D., Farias, E., Randolph, C. E., Purwin, T. J., McGrath, D., Charpentier, T. H., Zhang, L., Wu, S., Terai, M., Sato, T. et al. (2019). Effects of Oncogenic Galpha(q) and Galpha(11) Inhibition by FR900359 in uveal melanoma. *Mol. Cancer Res.* 17, 963-973. 10.1158/1541-7786.MCR-18-0574 30567972 PMC6445713

[DEV204396C45] Lawson, N. D. and Weinstein, B. M. (2002). In vivo imaging of embryonic vascular development using transgenic zebrafish. *Dev. Biol.* 248, 307-318. 10.1006/dbio.2002.0711 12167406

[DEV204396C46] Le Douarin, N. and Kalcheim, C. (1999). *The Neural Crest*. Cambridge, NY, USA: Cambridge University Press.

[DEV204396C47] Lei, R., Zhang, K., Wei, Y., Chen, M., Weinstein, L. S., Hong, Y., Zhu, M., Li, H. and Li, H. (2016). G-Protein alpha-subunit gsalpha is required for craniofacial morphogenesis. *PLoS ONE* 11, e0147535. 10.1371/journal.pone.0147535 26859889 PMC4747491

[DEV204396C48] Madeira, F., Park, Y. M., Lee, J., Buso, N., Gur, T., Madhusoodanan, N., Basutkar, P., Tivey, A. R. N., Potter, S. C., Finn, R. D. et al. (2019). The EMBL-EBI search and sequence analysis tools APIs in 2019. *Nucleic Acids Res.* 47, W636-W641. 10.1093/nar/gkz268 30976793 PMC6602479

[DEV204396C49] Malbon, C. C. (2005). G proteins in development. *Nat. Rev. Mol. Cell Biol.* 6, 689-701. 10.1038/nrm1716 16231420

[DEV204396C50] Marivin, A., Leyme, A., Parag-Sharma, K., DiGiacomo, V., Cheung, A. Y., Nguyen, L. T., Dominguez, I. and Garcia-Marcos, M. (2016). Dominant-negative Galpha subunits are a mechanism of dysregulated heterotrimeric G protein signaling in human disease. *Sci. Signal.* 9, ra37. 10.1126/scisignal.aad2429 27072656 PMC4870087

[DEV204396C51] Masuho, I., Kise, R., Gainza, P., Von Moo, E., Li, X., Tany, R., Wakasugi-Masuho, H., Correia, B. E. and Martemyanov, K. A. (2023). Rules and mechanisms governing G protein coupling selectivity of GPCRs. *Cell Rep.* 42, 113173. 10.1016/j.celrep.2023.113173 37742189 PMC10842385

[DEV204396C52] Meinecke, L., Sharma, P. P., Du, H., Zhang, L., Nie, Q. and Schilling, T. F. (2018). Modeling craniofacial development reveals spatiotemporal constraints on robust patterning of the mandibular arch. *PLoS Comput. Biol.* 14, e1006569. 10.1371/journal.pcbi.1006569 30481168 PMC6258504

[DEV204396C53] Miller, C. T. and Kimmel, C. B. (2001). Morpholino phenocopies of endothelin 1 (sucker) and other anterior arch class mutations. *Genesis* 30, 186-187. 10.1002/gene.1061 11477704

[DEV204396C54] Miller, C. T., Schilling, T. F., Lee, K., Parker, J. and Kimmel, C. B. (2000). sucker encodes a zebrafish Endothelin-1 required for ventral pharyngeal arch development. *Development* 127, 3815-3828. 10.1242/dev.127.17.3815 10934026

[DEV204396C55] Miller, C. T., Yelon, D., Stainier, D. Y. and Kimmel, C. B. (2003). Two endothelin 1 effectors, hand2 and bapx1, pattern ventral pharyngeal cartilage and the jaw joint. *Development* 130, 1353-1365. 10.1242/dev.00339 12588851

[DEV204396C56] Miller, C. T., Swartz, M. E., Khuu, P. A., Walker, M. B., Eberhart, J. K. and Kimmel, C. B. (2007). mef2ca is required in cranial neural crest to effect Endothelin1 signaling in zebrafish. *Dev. Biol.* 308, 144-157. 10.1016/j.ydbio.2007.05.018 17574232 PMC2148033

[DEV204396C57] Mitchell, J. M., Sucharov, J., Pulvino, A. T., Brooks, E. P., Gillen, A. E. and Nichols, J. T. (2021). The alx3 gene shapes the zebrafish neurocranium by regulating frontonasal neural crest cell differentiation timing. *Development* 148, dev197483. 10.1242/dev.197483 33741714 PMC8077506

[DEV204396C58] Nair, S., Li, W., Cornell, R. and Schilling, T. F. (2007). Requirements for Endothelin type-A receptors and Endothelin-1 signaling in the facial ectoderm for the patterning of skeletogenic neural crest cells in zebrafish. *Development* 134, 335-345. 10.1242/dev.02704 17166927

[DEV204396C60] Nestorowa, S., Hamey, F. K., Pijuan Sala, B., Diamanti, E., Shepherd, M., Laurenti, E., Wilson, N. K., Kent, D. G. and Gottgens, B. (2016). A single-cell resolution map of mouse hematopoietic stem and progenitor cell differentiation. *Blood* 128, e20-e31. 10.1182/blood-2016-05-716480 27365425 PMC5305050

[DEV204396C61] Neuhauss, S. C., Solnica-Krezel, L., Schier, A. F., Zwartkruis, F., Stemple, D. L., Malicki, J., Abdelilah, S., Stainier, D. Y. and Driever, W. (1996). Mutations affecting craniofacial development in zebrafish. *Development* 123, 357-367. 10.1242/dev.123.1.357 9007255

[DEV204396C62] Nishimura, A., Kitano, K., Takasaki, J., Taniguchi, M., Mizuno, N., Tago, K., Hakoshima, T. and Itoh, H. (2010). Structural basis for the specific inhibition of heterotrimeric Gq protein by a small molecule. *Proc. Natl. Acad. Sci. USA* 107, 13666-13671. 10.1073/pnas.1003553107 20639466 PMC2922266

[DEV204396C63] Offermanns, S., Zhao, L. P., Gohla, A., Sarosi, I., Simon, M. I. and Wilkie, T. M. (1998). Embryonic cardiomyocyte hypoplasia and craniofacial defects in G alpha q/G alpha 11-mutant mice. *EMBO J.* 17, 4304-4312. 10.1093/emboj/17.15.4304 9687499 PMC1170764

[DEV204396C64] Ohmoto, M., Okada, S., Nakamura, S., Abe, K. and Matsumoto, I. (2011). Mutually exclusive expression of Galphaia and Galpha14 reveals diversification of taste receptor cells in zebrafish. *J. Comp. Neurol.* 519, 1616-1629. 10.1002/cne.22589 21452212 PMC3394409

[DEV204396C65] Oka, Y. and Korsching, S. I. (2011). Shared and unique G alpha proteins in the zebrafish versus mammalian senses of taste and smell. *Chem. Senses* 36, 357-365. 10.1093/chemse/bjq138 21242316

[DEV204396C66] Okashah, N., Wan, Q., Ghosh, S., Sandhu, M., Inoue, A., Vaidehi, N. and Lambert, N. A. (2019). Variable G protein determinants of GPCR coupling selectivity. *Proc. Natl. Acad. Sci. USA* 116, 12054-12059. 10.1073/pnas.1905993116 31142646 PMC6575158

[DEV204396C67] Oldham, W. M. and Hamm, H. E. (2008). Heterotrimeric G protein activation by G-protein-coupled receptors. *Nat. Rev. Mol. Cell Biol.* 9, 60-71. 10.1038/nrm2299 18043707

[DEV204396C68] Onken, M. D., Makepeace, C. M., Kaltenbronn, K. M., Kanai, S. M., Todd, T. D., Wang, S., Broekelmann, T. J., Rao, P. K., Cooper, J. A. and Blumer, K. J. (2018). Targeting nucleotide exchange to inhibit constitutively active G protein alpha subunits in cancer cells. *Sci. Signal.* 11, eaao6852. 10.1126/scisignal.aao6852 30181242 PMC6279241

[DEV204396C69] Ozeki, H., Kurihara, Y., Tonami, K., Watatani, S. and Kurihara, H. (2004). Endothelin-1 regulates the dorsoventral branchial arch patterning in mice. *Mech. Dev.* 121, 387-395. 10.1016/j.mod.2004.02.002 15110048

[DEV204396C70] Pires-daSilva, A. and Sommer, R. J. (2003). The evolution of signalling pathways in animal development. *Nat. Rev. Genet.* 4, 39-49. 10.1038/nrg977 12509752

[DEV204396C71] Plummer, N. W., Spicher, K., Malphurs, J., Akiyama, H., Abramowitz, J., Nurnberg, B. and Birnbaumer, L. (2012). Development of the mammalian axial skeleton requires signaling through the Galpha(i) subfamily of heterotrimeric G proteins. *Proc. Natl. Acad. Sci. USA* 109, 21366-21371. 10.1073/pnas.1219810110 23236180 PMC3535641

[DEV204396C72] Pritchard, A. B., Kanai, S. M., Krock, B., Schindewolf, E., Oliver-Krasinski, J., Khalek, N., Okashah, N., Lambert, N. A., Tavares, A. L. P., Zackai, E. et al. (2020). Loss-of-function of Endothelin receptor type A results in Oro-Oto-Cardiac syndrome. *Am. J. Med. Genet. A* 182, 1104-1116. 10.1002/ajmg.a.61531 32133772 PMC7202054

[DEV204396C73] Randlett, O., Wee, C. L., Naumann, E. A., Nnaemeka, O., Schoppik, D., Fitzgerald, J. E., Portugues, R., Lacoste, A. M., Riegler, C., Engert, F. et al. (2015). Whole-brain activity mapping onto a zebrafish brain atlas. *Nat. Methods* 12, 1039-1046. 10.1038/nmeth.3581 26778924 PMC4710481

[DEV204396C74] Rieder, M. J., Green, G. E., Park, S. S., Stamper, B. D., Gordon, C. T., Johnson, J. M., Cunniff, C. M., Smith, J. D., Emery, S. B., Lyonnet, S. et al. (2012). A human homeotic transformation resulting from mutations in PLCB4 and GNAI3 causes auriculocondylar syndrome. *Am. J. Hum. Genet.* 90, 907-914. 10.1016/j.ajhg.2012.04.002 22560091 PMC3376493

[DEV204396C75] Ruest, L. B. and Clouthier, D. E. (2009). Elucidating timing and function of endothelin-A receptor signaling during craniofacial development using neural crest cell-specific gene deletion and receptor antagonism. *Dev. Biol.* 328, 94-108. 10.1016/j.ydbio.2009.01.005 19185569 PMC2821796

[DEV204396C76] Ruest, L. B., Xiang, X., Lim, K. C., Levi, G. and Clouthier, D. E. (2004). Endothelin-A receptor-dependent and -independent signaling pathways in establishing mandibular identity. *Development* 131, 4413-4423. 10.1242/dev.01291 15306564 PMC2818681

[DEV204396C77] Sato, T., Kawamura, Y., Asai, R., Amano, T., Uchijima, Y., Dettlaff-Swiercz, D. A., Offermanns, S., Kurihara, Y. and Kurihara, H. (2008a). Recombinase-mediated cassette exchange reveals the selective use of Gq/G11-dependent and -independent endothelin 1/endothelin type A receptor signaling in pharyngeal arch development. *Development* 135, 755-765. 10.1242/dev.012708 18199583

[DEV204396C78] Sato, T., Kurihara, Y., Asai, R., Kawamura, Y., Tonami, K., Uchijima, Y., Heude, E., Ekker, M., Levi, G. and Kurihara, H. (2008b). An endothelin-1 switch specifies maxillomandibular identity. *Proc. Natl. Acad. Sci. USA* 105, 18806-18811. 10.1073/pnas.0807345105 19017795 PMC2596216

[DEV204396C79] Schindelin, J., Arganda-Carreras, I., Frise, E., Kaynig, V., Longair, M., Pietzsch, T., Preibisch, S., Rueden, C., Saalfeld, S., Schmid, B. et al. (2012). Fiji: an open-source platform for biological-image analysis. *Nat. Methods* 9, 676-682. 10.1038/nmeth.2019 22743772 PMC3855844

[DEV204396C80] Schlegel, J. G., Tahoun, M., Seidinger, A., Voss, J. H., Kuschak, M., Kehraus, S., Schneider, M., Matthey, M., Fleischmann, B. K., Konig, G. M. et al. (2021). Macrocyclic Gq protein inhibitors FR900359 and/or YM-254890-fit for translation? *ACS Pharmacol. Transl. Sci.* 4, 888-897. 10.1021/acsptsci.1c00021 33860209 PMC8033771

[DEV204396C81] Schrage, R., Schmitz, A. L., Gaffal, E., Annala, S., Kehraus, S., Wenzel, D., Bullesbach, K. M., Bald, T., Inoue, A., Shinjo, Y. et al. (2015). The experimental power of FR900359 to study Gq-regulated biological processes. *Nat. Commun.* 6, 10156. 10.1038/ncomms10156 26658454 PMC4682109

[DEV204396C82] Shibata, T., Kawakami, K., Kawana, H., Aoki, J. and Inoue, A. (2022). Phenotypic evaluation of constitutive GPCR/G-protein signaling in zebrafish embryos and larvae. *Biochem. Biophys. Res. Commun.* 602, 70-76. 10.1016/j.bbrc.2022.02.098 35255436

[DEV204396C83] Stainier, D. Y., Fouquet, B., Chen, J. N., Warren, K. S., Weinstein, B. M., Meiler, S. E., Mohideen, M. A., Neuhauss, S. C., Solnica-Krezel, L., Schier, A. F. et al. (1996). Mutations affecting the formation and function of the cardiovascular system in the zebrafish embryo. *Development* 123, 285-292. 10.1242/dev.123.1.285 9007248

[DEV204396C84] Stenzel, A., Mumme-Monheit, A., Sucharov, J., Walker, M., Mitchell, J. M., Appel, B. and Nichols, J. T. (2022). Distinct and redundant roles for zebrafish her genes during mineralization and craniofacial patterning. *Front. Endocrinol. (Lausanne)* 13, 1033843. 10.3389/fendo.2022.1033843 36578958 PMC9791542

[DEV204396C85] Sucharov, J., Ray, K., Brooks, E. P. and Nichols, J. T. (2019). Selective breeding modifies mef2ca mutant incomplete penetrance by tuning the opposing Notch pathway. *PLoS Genet.* 15, e1008507. 10.1371/journal.pgen.1008507 31790396 PMC6907857

[DEV204396C86] Takasaki, J., Saito, T., Taniguchi, M., Kawasaki, T., Moritani, Y., Hayashi, K. and Kobori, M. (2004). A novel Galphaq/11-selective inhibitor. *J. Biol. Chem.* 279, 47438-47445. 10.1074/jbc.M408846200 15339913

[DEV204396C108] Takeuchi, M., Yamaguchi, S., Sakakibara, Y., Hayashi, T., Matsuda, K., Hara, Y., Tanegashima, C., Shimizu, T., Kuraku, S. and Hibi, M. (2017). Gene expression profiling of granule cells and Purkinje cells in the zebrafish cerebellum. *J. Comp. Neurol.* 525, 1558-1585. 10.1002/cne.24114 27615194

[DEV204396C87] Talbot, J. C., Johnson, S. L. and Kimmel, C. B. (2010). hand2 and Dlx genes specify dorsal, intermediate and ventral domains within zebrafish pharyngeal arches. *Development* 137, 2507-2517. 10.1242/dev.049700 20573696 PMC2927700

[DEV204396C88] Tang, W. and Bronner, M. E. (2020). Neural crest lineage analysis: from past to future trajectory. *Development* 147, dev193193. 10.1242/dev.193193 33097550 PMC7595686

[DEV204396C89] Tavares, A. L. and Clouthier, D. E. (2015). Cre recombinase-regulated Endothelin1 transgenic mouse lines: novel tools for analysis of embryonic and adult disorders. *Dev. Biol.* 400, 191-201. 10.1016/j.ydbio.2015.01.027 25725491 PMC4385399

[DEV204396C90] Tavares, A. L., Garcia, E. L., Kuhn, K., Woods, C. M., Williams, T. and Clouthier, D. E. (2012). Ectodermal-derived Endothelin1 is required for patterning the distal and intermediate domains of the mouse mandibular arch. *Dev. Biol.* 371, 47-56. 10.1016/j.ydbio.2012.08.003 22902530 PMC3470875

[DEV204396C91] Tavares, A. L. P., Cox, T. C., Maxson, R. M., Ford, H. L. and Clouthier, D. E. (2017). Negative regulation of endothelin signaling by SIX1 is required for proper maxillary development. *Development* 144, 2021-2031. 10.1242/dev.145144 28455376 PMC5482985

[DEV204396C92] Teng, C. S., Yen, H. Y., Barske, L., Smith, B., Llamas, J., Segil, N., Go, J., Sanchez-Lara, P. A., Maxson, R. E. and Crump, J. G. (2017). Requirement for Jagged1-Notch2 signaling in patterning the bones of the mouse and human middle ear. *Sci. Rep.* 7, 2497. 10.1038/s41598-017-02574-7 28566723 PMC5451394

[DEV204396C93] Thisse, C. and Thisse, B. (2008). High-resolution in situ hybridization to whole-mount zebrafish embryos. *Nat. Protoc.* 3, 59-69. 10.1038/nprot.2007.514 18193022

[DEV204396C94] Thomas, T., Kurihara, H., Yamagishi, H., Kurihara, Y., Yazaki, Y., Olson, E. N. and Srivastava, D. (1998). A signaling cascade involving endothelin-1, dHAND and msx1 regulates development of neural-crest-derived branchial arch mesenchyme. *Development* 125, 3005-3014. 10.1242/dev.125.16.3005 9671575

[DEV204396C95] Van Raamsdonk, C. D., Bezrookove, V., Green, G., Bauer, J., Gaugler, L., O'Brien, J. M., Simpson, E. M., Barsh, G. S. and Bastian, B. C. (2009). Frequent somatic mutations of GNAQ in uveal melanoma and blue naevi. *Nature* 457, 599-602. 10.1038/nature07586 19078957 PMC2696133

[DEV204396C96] Vieux-Rochas, M., Mantero, S., Heude, E., Barbieri, O., Astigiano, S., Couly, G., Kurihara, H., Levi, G. and Merlo, G. R. (2010). Spatio-temporal dynamics of gene expression of the Edn1-Dlx5/6 pathway during development of the lower jaw. *Genesis* 48, 262-373. 10.1002/dvg.20625 20333701

[DEV204396C97] Walker, M. B. and Kimmel, C. B. (2007). A two-color acid-free cartilage and bone stain for zebrafish larvae. *Biotech. Histochem.* 82, 23-28. 10.1080/10520290701333558 17510811

[DEV204396C98] Walker, M. B., Miller, C. T., Coffin Talbot, J., Stock, D. W. and Kimmel, C. B. (2006). Zebrafish furin mutants reveal intricacies in regulating Endothelin1 signaling in craniofacial patterning. *Dev. Biol.* 295, 194-205. 10.1016/j.ydbio.2006.03.028 16678149

[DEV204396C99] Walker, M. B., Miller, C. T., Swartz, M. E., Eberhart, J. K. and Kimmel, C. B. (2007). phospholipase C, beta 3 is required for Endothelin1 regulation of pharyngeal arch patterning in zebrafish. *Dev. Biol.* 304, 194-207. 10.1016/j.ydbio.2006.12.027 17239364 PMC1906931

[DEV204396C100] Weinschutz Mendes, H., Taktek, M., Duret, T. and Ekker, M. (2020). Expression of dlx genes in the normal and regenerating brain of adult zebrafish. *PLoS One* 15, e0229549. 10.1371/journal.pone.0229549 32497078 PMC7272068

[DEV204396C101] Westerfield, M. (2007). *The Zebrafish Book. A Guide for the Laboratory Use of Zebrafish (Danio rerio)*, 5th edn. Eugene: University of Oregon Press.

[DEV204396C102] Wettschureck, N. and Offermanns, S. (2005). Mammalian G proteins and their cell type specific functions. *Physiol. Rev.* 85, 1159-1204. 10.1152/physrev.00003.2005 16183910

[DEV204396C103] Yanagisawa, H., Hammer, R. E., Richardson, J. A., Williams, S. C., Clouthier, D. E. and Yanagisawa, M. (1998a). Role of Endothelin-1/Endothelin-A receptor-mediated signaling pathway in the aortic arch patterning in mice. *J. Clin. Invest.* 102, 22-33. 10.1172/JCI2698 9649553 PMC509061

[DEV204396C104] Yanagisawa, H., Yanagisawa, M., Kapur, R. P., Richardson, J. A., Williams, S. C., Clouthier, D. E., de Wit, D., Emoto, N. and Hammer, R. E. (1998b). Dual genetic pathways of endothelin-mediated intercellular signaling revealed by targeted disruption of endothelin converting enzyme-1 gene. *Development* 125, 825-836. 10.1242/dev.125.5.825 9449665

[DEV204396C105] Zuniga, E., Stellabotte, F. and Crump, J. G. (2010). Jagged-Notch signaling ensures dorsal skeletal identity in the vertebrate face. *Development* 137, 1843-1852. 10.1242/dev.049056 20431122 PMC2867320

[DEV204396C106] Zuniga, E., Rippen, M., Alexander, C., Schilling, T. F. and Crump, J. G. (2011). Gremlin 2 regulates distinct roles of BMP and Endothelin 1 signaling in dorsoventral patterning of the facial skeleton. *Development* 138, 5147-5156. 10.1242/dev.067785 22031546 PMC3210496

