# Supplementary material for: The Gq/11 family of Gα subunits is necessary and sufficient for lower jaw development
Source: Development. 2025 Apr 17;152(8):dev204396. doi: 10.1242/dev.204396 (PMC12045641; doi:10.1242/dev.204396)
Supplement: Supplementary information [file develop-152-204396-s1.pdf]

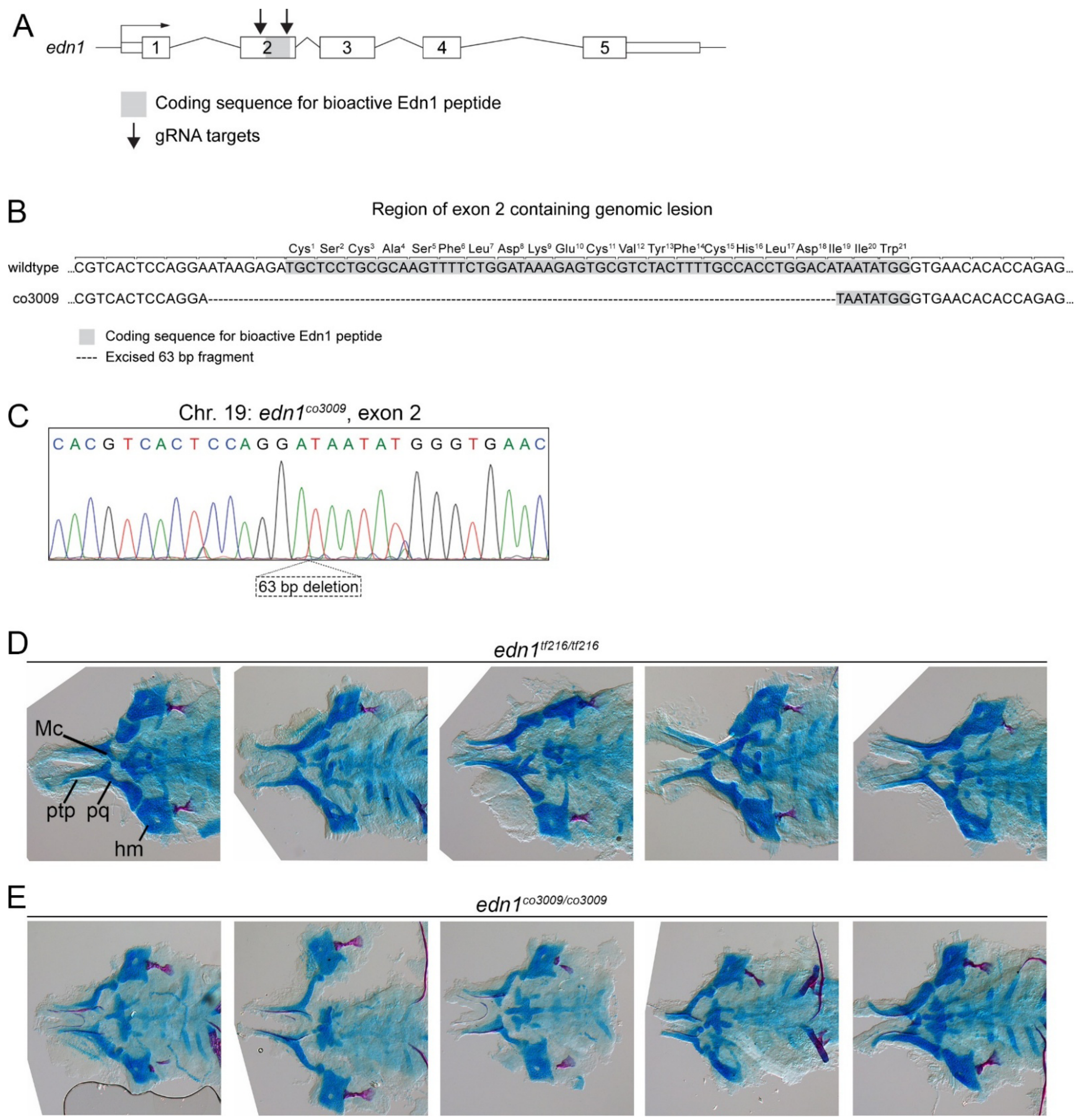

Fig. S1. Targeting strategy for new *edn1* allele and characterization of genomic feature and phenotype. (A) Schematic of gene locus for *edn1* and targeting sites for sgRNAs (arrows). The coding sequence for the bioactive portion of Edn1 is in exon2 (in grey). **(B)** The co3009 allele contains a 63 bp in-frame deletion in exon 2, excising a majority of the coding sequence for the bioactive peptide. Excised nucleotides are indicated with dashed lines. The amino acids for the bioactive Edn1 peptide are labeled. The open reading frame is indicated with brackets. **(C)** Sanger sequencing traces from homozygous mutant animals confirm the genomic lesion. **(D,E)** Craniofacial phenotypes are indistinguishable between larvae homozygous for the *tf216/sucker* allele and the *co3009* deletion allele**.** Five representative flat-mounts of the viscerocranium at 6 dpf are shown for (D) *edn1*$^{tf216/tf216}$ (n=63) and (E) *edn1*$^{co3009/co3009}$ (n=41) larvae.

Development • Supplementary information

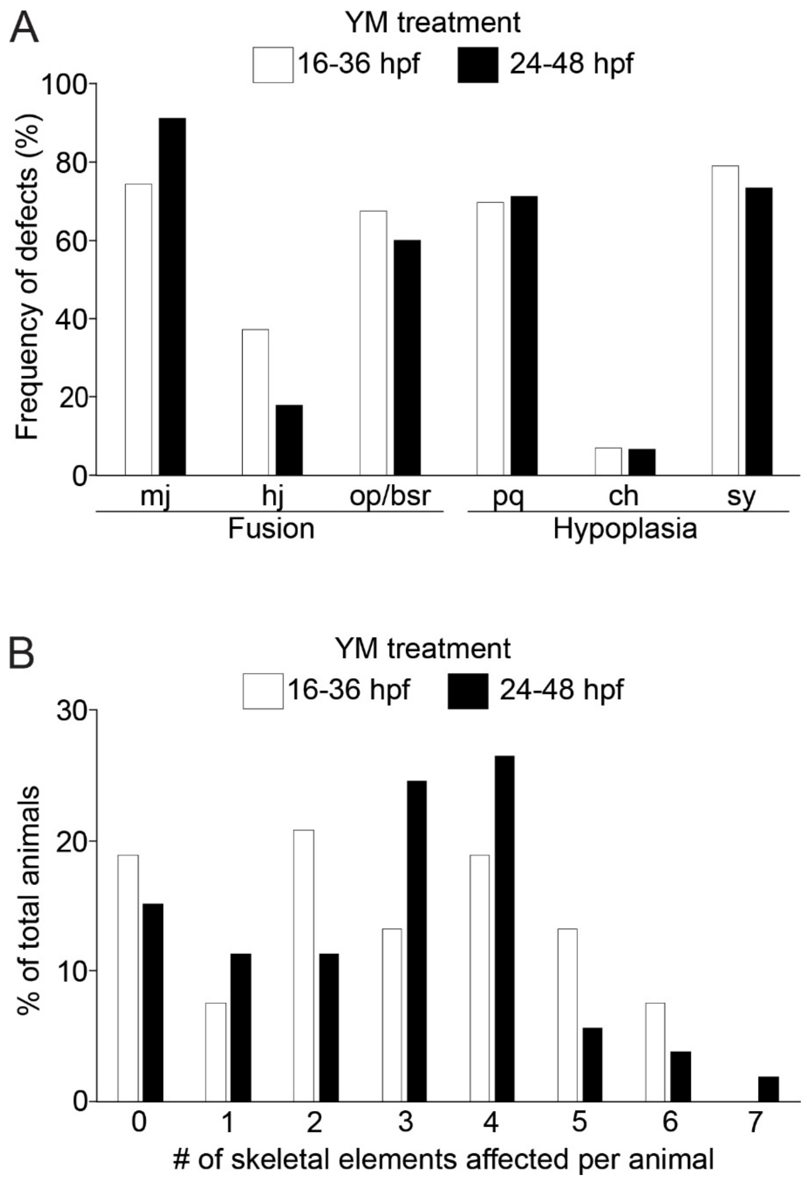

Fig. S2. Frequency and overall severity of defects in YM-treated larvae. Shown are results for embryos treated with YM between 16-36 hpf vs 24-48 hpf. **(A)** Frequency of defects in specific skeletal elements for all larvae. (p=0.1, chi-square test) **(B)** Overall severity of defects, expressed as the number of skeletal elements affected per individual larva.

Development • Supplementary information

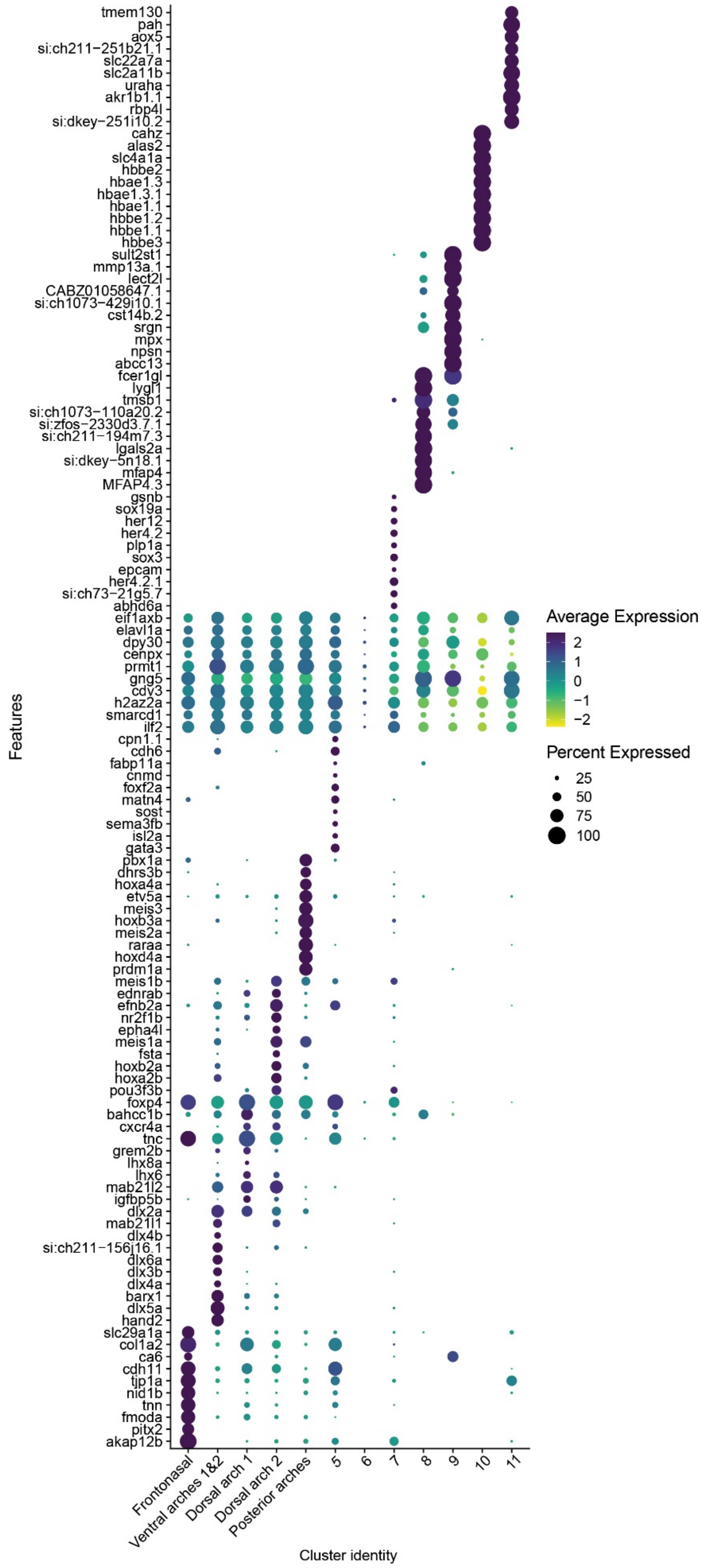

Fig. S3. Dot plot analysis of marker genes. Shown are top ten genes per cluster based on adjusted p value.

Development • Supplementary information

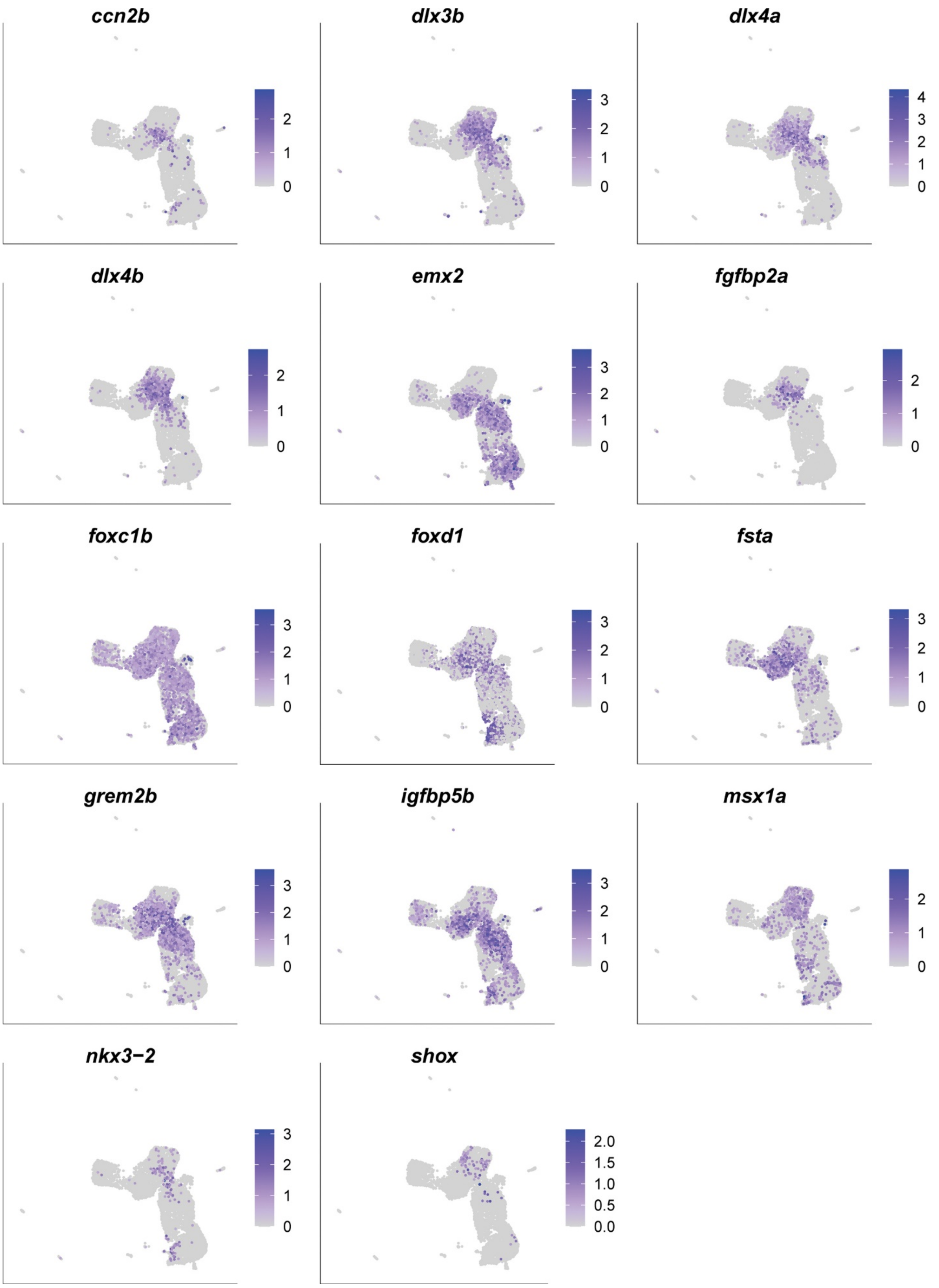

Fig. S4. Feature maps of marker genes used to define the intermediate domain. The selected marker genes have been experimentally verified. Scale is average expression.

Development • Supplementary information

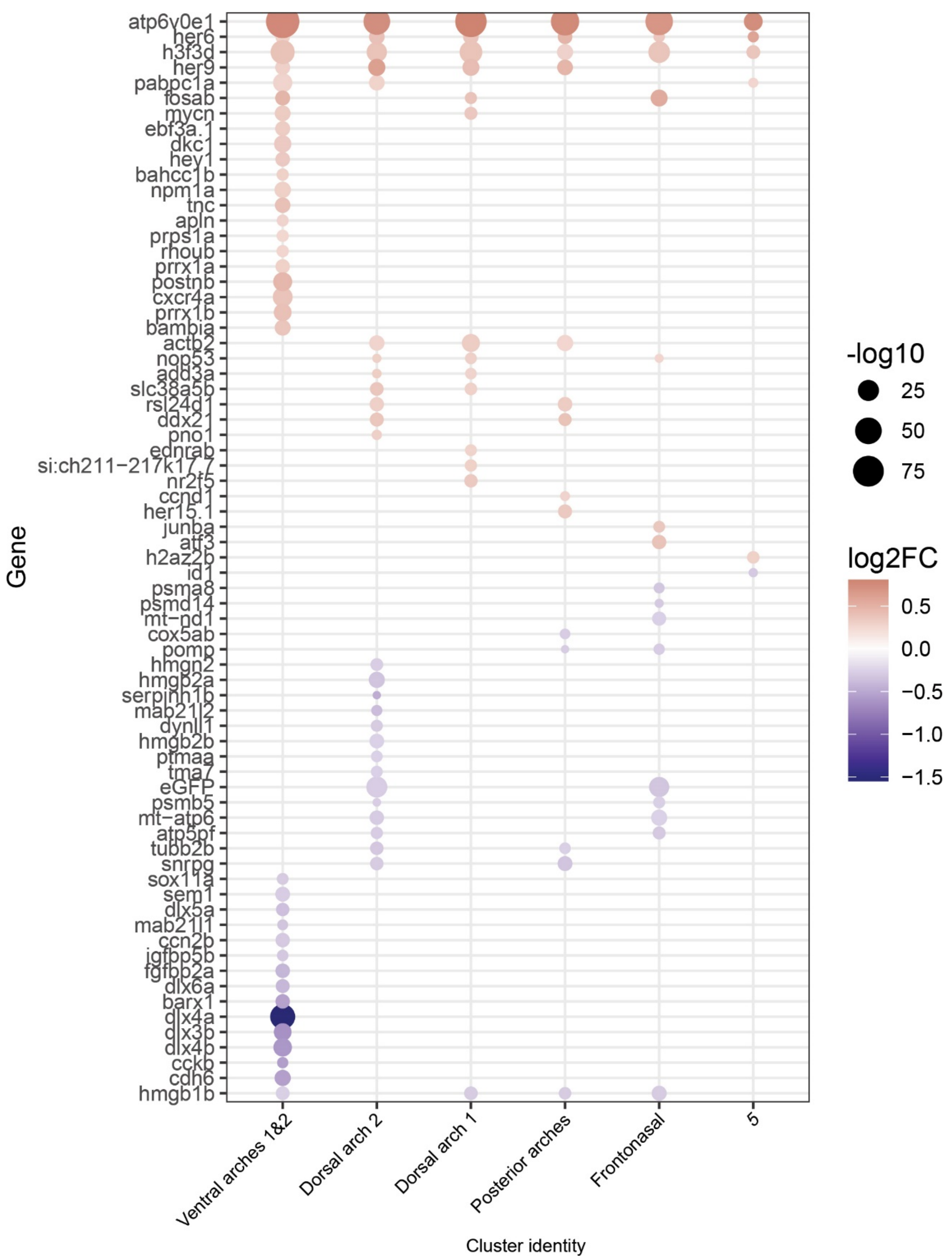

Fig. S5. Dot plot of differentially expressed genes. All differentially expressed genes are based on adjusted p values in YM-treated samples relative to DMSO-treated control samples, across equivalent clusters. Differential expression is displayed as log2-fold change (scale bar).

Development • Supplementary information

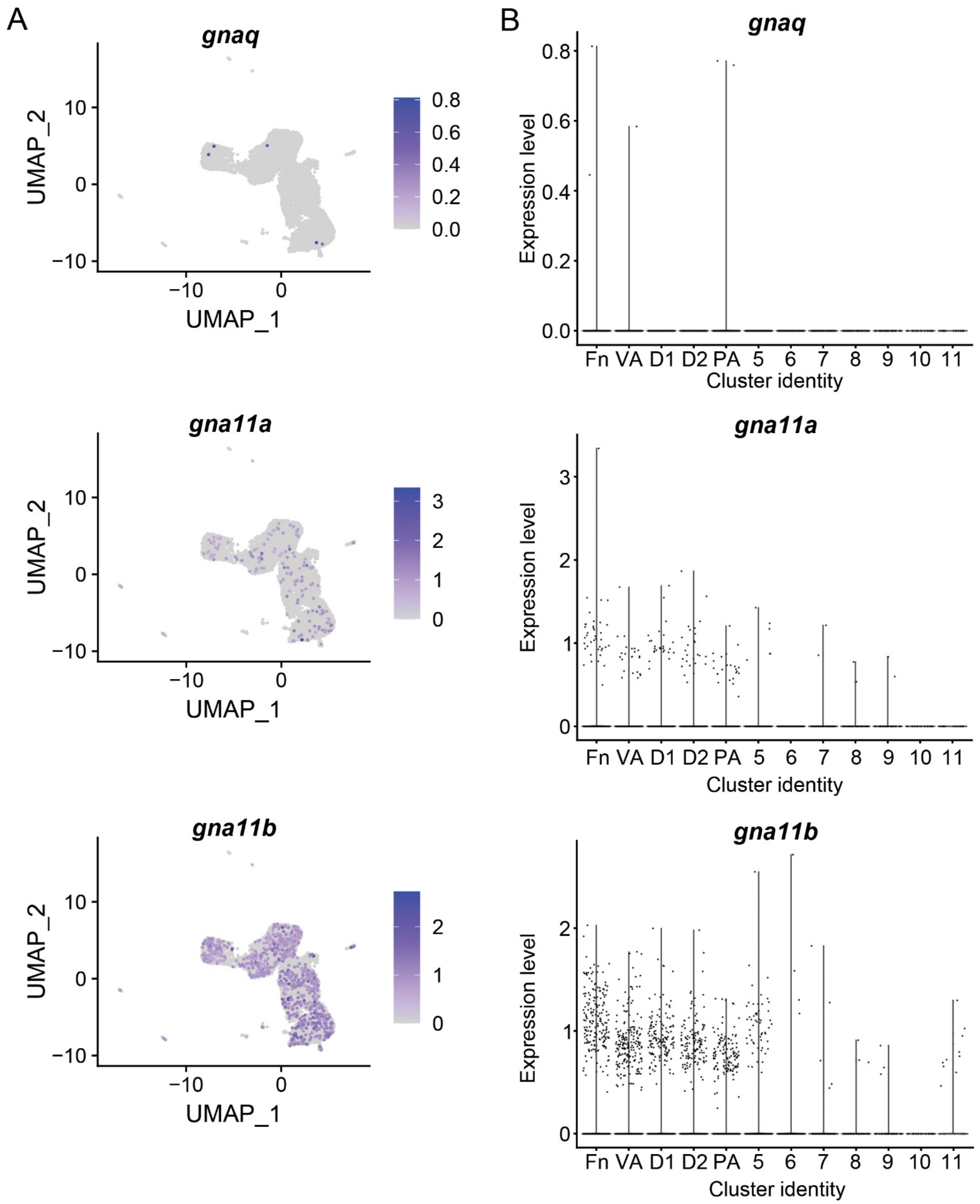

Fig. S6. *gnaq*, *gna11a* and *gna11b* are differentially expressed in cranial neural crest cells. Expression profiles for *gnaq, gna11a* and *gna11b* are shown in **(A)** UMAP plots and **(B)** Violin plots. Scale is average expression. D1; Dorsal arch 1, D2; Dorsal arch 2, Fn; Frontonasal, PA; Posterior arches, VA; Ventral arches 1&2

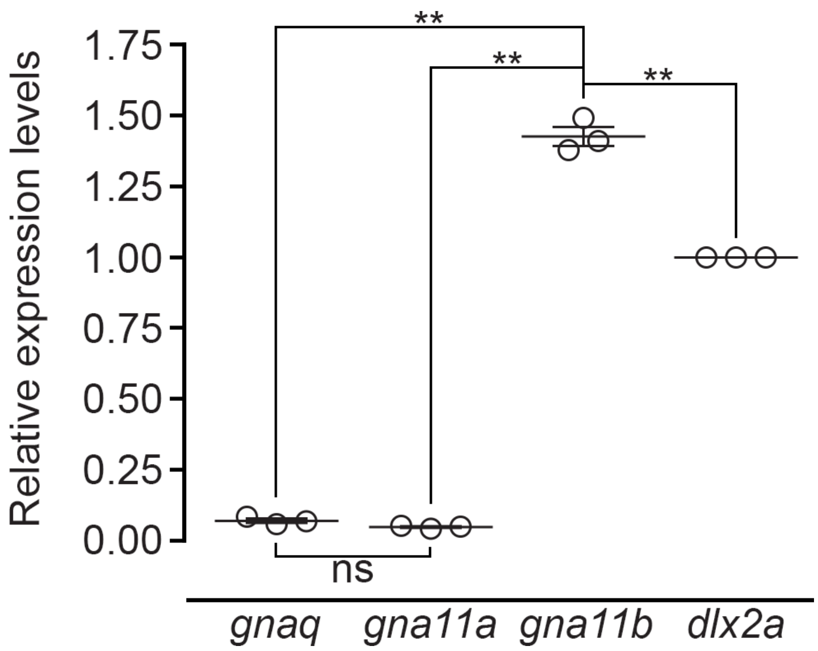

Fig. S7. Expression analysis of Gq/11 genes with qPCR. RNA isolated from whole heads of 28 hpf wild-type zebrafish embryos was analyzed by qPCR. Average expression levels of *gnaq*, *gna11a*, and *gna11b* are shown relative to *dlx2a.* The circles represent biological replicates, with one replicate consisting of RNA pooled from 40 embryo heads. Statistical significance was determined by an unpaired t-test (**; $p < 0.01$, ns; not significant). Error bars are SEM.

Development • Supplementary information

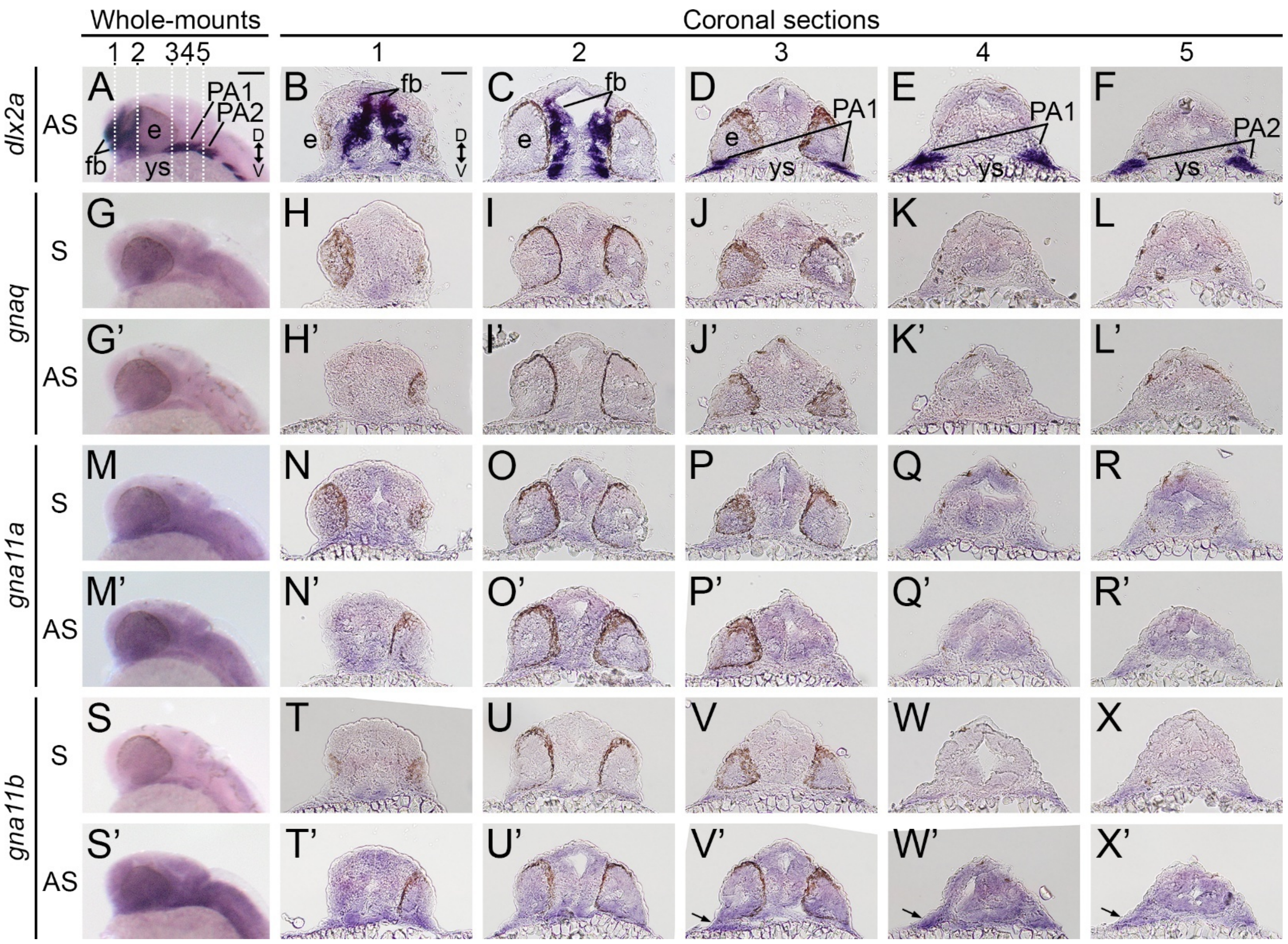

Fig. S8. Expression analysis of Gq/11 genes with in situ hybridization. 28 hpf wild-type zebrafish embryos processed for whole-mount in situ hybridization (A, G, G', M, M', S, S') were sectioned along the coronal plane (B-F, H-L, H'-L', N-R, N'-R', T-X, T'-X'). The vertical dashed lines numbered 1-5 in panel A indicate the axial positions of the five coronal sections shown. An antisense (AS) probe for *dlx2a* was used (A-F) to establish the location of pharyngeal arch 1 (PA1; D,E) and pharyngeal arch 2 (PA2; F) in the sections. Sense (S) probes are shown for *gnaq* (G-L), *gna11a* (M-R), and *gna11b* (S-X). Antisense (AS) probes are shown for *gnaq* (G'-L'), *gna11a* (M'-R'), *gna11b* (S'-X'). Arrows in V' and W' point to PA1. Arrow in X' point to PA2. Scale bar in A is 100 µm. Scale bar in B is 40 µm. D; dorsal, e; eye, fb; forebrain, V; ventral, ys, yolk sack

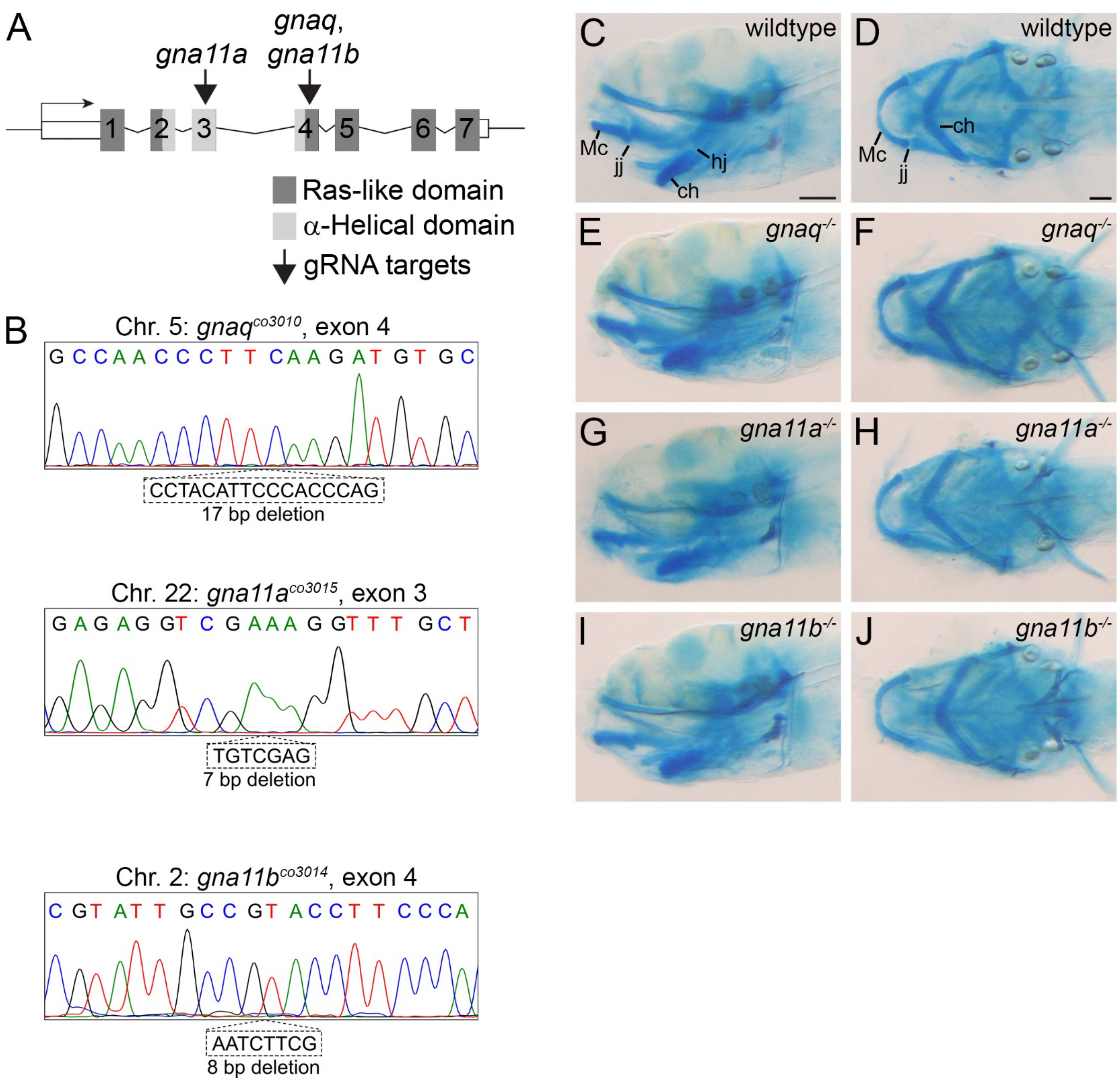

Fig. S9. Targeting strategy for CRISPR/Cas9 gene-editing and characterized alleles. (A) Schematic of a generalized gene locus for *gnaq, gna11a* and *gna11b*, and the targeting sites for sgRNAs. Numbers indicate exons. Exons encoding for the Ras-like and α-Helical domains are indicated. **(B)** Sanger sequencing traces from homozygous mutant animals confirm frameshift-causing deletions for the alleles *gnaq*^{co3010}, *gna11a*^{co3015} and *gna11b*^{co3014}. For all alleles, the respective deletions result in a premature stop codon in exon 4. **(C-J)** Whole-mount skeletal preparations, with eyes removed, of wildtype (C,D), *gnaq*$^{-/-}$ (E,F), *gna11a*$^{-/-}$ (G,H), and *gna11b*$^{-/-}$ (I,J) larvae in lateral (C,E,G,I) and ventral (D,F,H,J) views. All larvae are 6 dpf. Scale bar in (C) is 250 µm. Scale bar in (D) is 100 µm. ch, ceratohyal; hj, hyomandibular joint; jj, jaw joint; Mc, Meckel's cartilage.

Development • Supplementary information

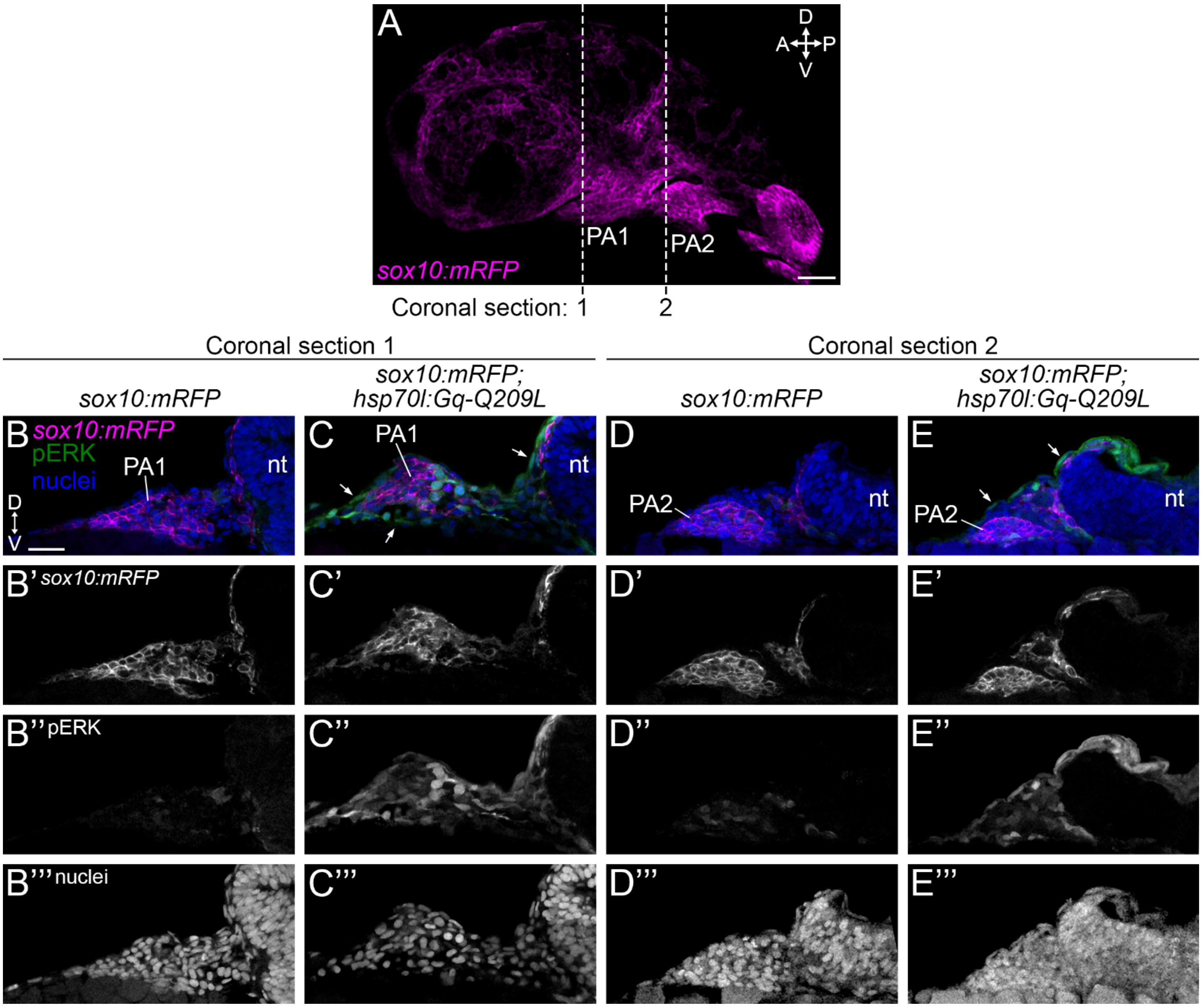

Fig. S10. Immunohistochemistry with a phosphorylated ERK (pERK) antibody on coronal sections from heat-shocked embryos. *sox10:mRFP;hsp70l:Gq-Q209L* and *sox10:mRFP* (sibling control) zebrafish embryos that were heat-shocked at 21 hpf and fixed three hours later at 24 hpf were sectioned along the coronal plane. **(A)** Lateral view of a sox10:mRFP zebrafish embryo at 24 hpf, with the vertical dashed lines indicating the coronal sections shown in B-E. Coronal section 1 captures pharyngeal arch 1 (PA1) (B,C), and Coronal section 2 captures pharyngeal arch 2 (PA2) (D, E). **(B,C,D,E)** Overlay of pseudo-colored sox10:mRFP (magenta), pERK (green), and nuclei (blue). The individual channels are shown in greyscale for *sox10:mRFP* (B',C',D',E'), pERK (B'',C'',D'',E'') and nuclei (B''',C''',D''',E'''). Sections for sibling control embryos (*sox10:mRFP*) are shown for PA1 (B, B,B'',B''') and PA2 (D,D',D'',D'''), and sections for *sox10:mRFP;hsp70l:Gq-Q209L* embryos are shown for PA1 (C,C',C'',C''') and PA2 (E,E',E'',E'''). Images are max projections of at least two Z-plane images. Shown are representative images from at least three animals per condition. Scale bar in A is 50 μm. Scale bar in B is 20 μm.

Development • Supplementary information

A

	Transgene (cardiac GFP expression)			
	Negative		Positive	
	n	**%**	***n***	**%**
edn1$^{+/+}$ *and edn1*$^{+/-}$	39	76.5	42	50.6
edn1$^{-/-}$	12	23.5	9	10.8
severe heart edema (all genotypes represented)	0	0	32	38.6
total	51	100	83	100

B

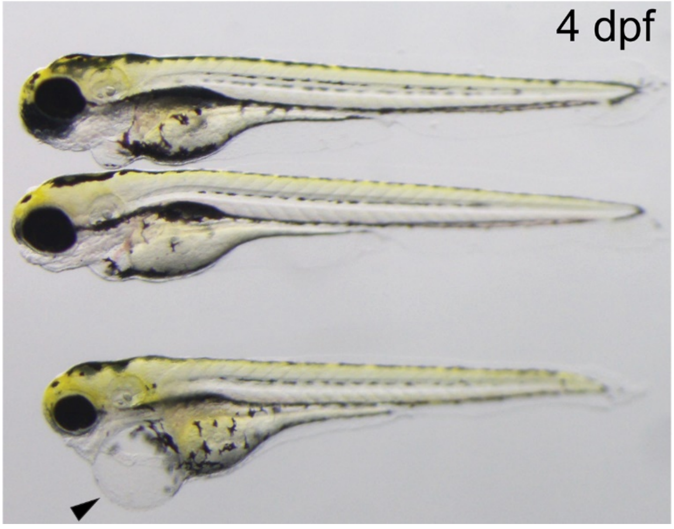

Fig. S11. Frequency and example of heart edema associated with the *hsp70l:Gq-Q209L* transgene. **(A)** Table showing the frequency of genotypes and heart edema observed from embryos generated from *edn1*$^{+/-}$ x *edn1*$^{+/-}$*;hsp70l:Gq-Q209L* crosses, which were subsequently heat-shocked. Heart edema was associated only with transgene-positive larvae, for all *edn1* genotypes (*edn1*$^{+/+}$, *edn1*$^{+/-}$, *edn1*$^{-/-}$). Larvae exhibiting heart edema (n=32) were excluded from the analysis. **(B)** Example of severe heart edema (arrow) associated with the *hsp70l:Gq-Q209L* transgene.

Table S1. Marker genes for clusters generated from the integrated dataset comprising DMSO- and YM-treated samples. The numbers in the "cluster" column correspond to the numbers in the UMAPs presented below. These UMAPs are identical to Figure 3B, though with clusters labeled with numbers rather than names of specific NCC populations. The names of the cranial NCC populations are indicated in the adjacent column labeled "cell population".

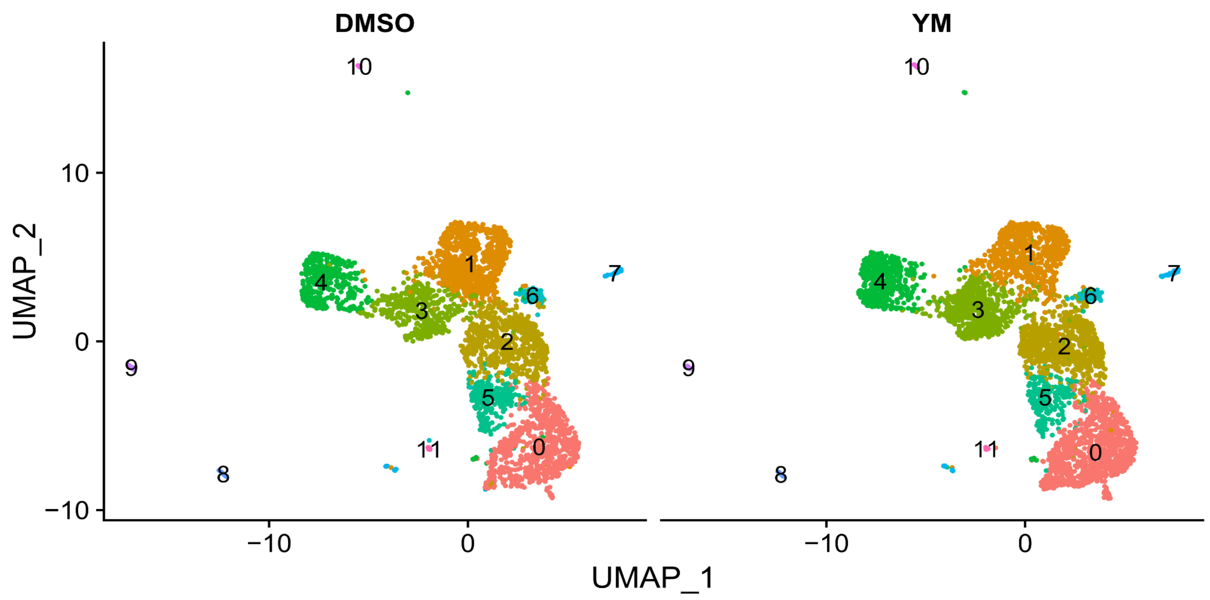

Available for download at
https://journals.biologists.com/dev/article-lookup/doi/10.1242/dev.204396#supplementary-data

Table S2. Differentially expressed genes in DMSO- versus YM-treated samples. Differential expression analysis was performed between equivalent clusters in DMSO- and YM-treated samples. Cluster identities are indicated by the numbers and names in the "cluster" and "cell population" columns, respectively, which correspond to the UMAPs shown below. These UMAPs are identical to those in Figure 3B, with clusters labeled with numbers rather than names of specific NCC populations. The "avg_log2FC" values indicate differential expression in DMSO-treated samples relative to YM-treated samples.

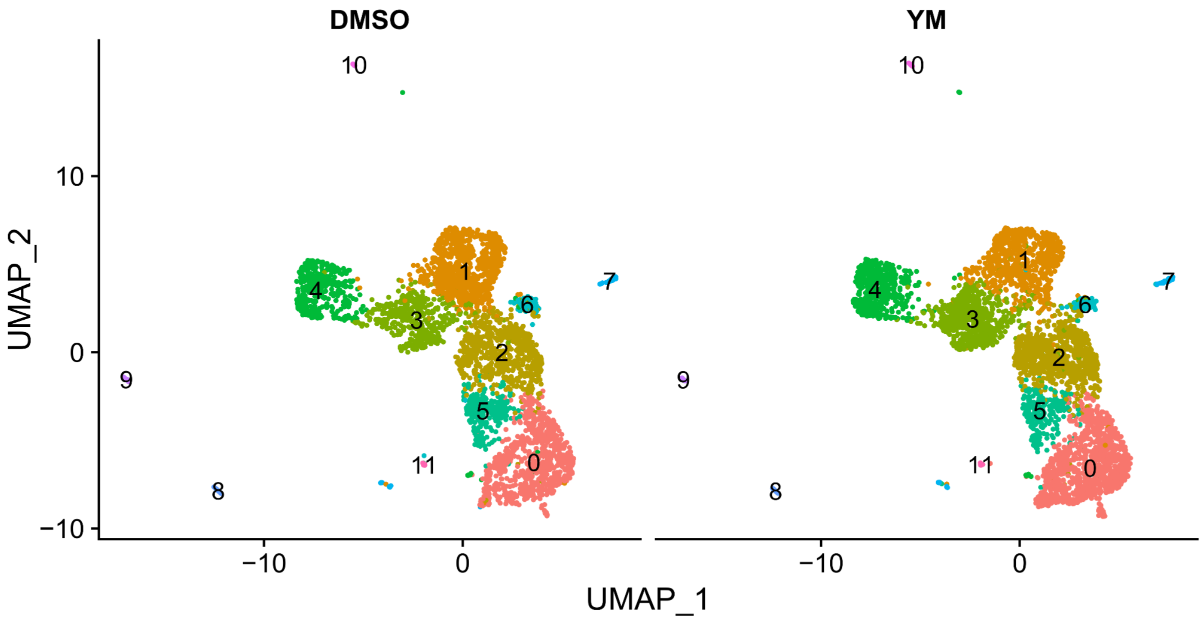

Available for download at
https://journals.biologists.com/dev/article-lookup/doi/10.1242/dev.204396#supplementary-data

Table S3. Table of predicted and observed genotypes obtained from crossing *gnaq*$^{+/-}$;*gna11a*$^{+/-}$;*gna11b*$^{+/-}$ animals. Values represent the sum of three independent clutches. Differences in predicted and observed values are not statistically significant (*p*=0.4, chi-square test)

Genotype (*gnaq; gna11a; gna11b*)	Pedicted # per Genotype	Observed # per Genotype	Predicted Frequency (%)	Observed Frequency (%)
-/- ; -/- ; -/-	5.24	10.00	1.56	2.98
-/- ; -/- ; +/+	5.24	5.00	1.56	1.49
-/- ; +/+ ; -/-	5.24	3.00	1.56	0.89
-/- ; +/+ ; +/+	5.24	5.00	1.56	1.49
- /- ; -/- ; +/-	10.50	17.00	3.13	5.06
-/- ; +/- ; -/-	10.50	12.00	3.13	3.57
-/- ; +/- ; +/-	21.00	34.00	6.25	10.12
-/- ; +/- ; +/+	10.50	10.00	3.13	2.98
-/- ; +/+ ; +/-	10.50	10.00	3.13	2.98
+/- ; -/- ; -/-	10.50	10.00	3.13	2.98
+/- ; -/- ; +/+	10.50	12.00	3.13	3.57
+/- ; -/- ; +/-	21.00	18.00	6.25	5.36
+/- ; +/- ; -/-	21.00	18.00	6.25	5.36
+/- ; +/- ; +/-	42.00	43.00	12.50	12.80
+/- ; +/- ; +/+	21.00	21.00	6.25	6.25
+/- ; +/+ ; +/-	21.00	19.00	6.25	5.65
+/+ ; -/- ; +/-	10.50	7.00	3.13	2.08
+/- ; +/+ ; -/-	10.50	9.00	3.13	2.68
+/+ ; +/- ; -/-	10.50	8.00	3.13	2.38
+/- ; +/+ ; +/+	10.50	6.00	3.13	1.79
+/+ ; +/- ; +/-	21.00	26.00	6.25	7.74
+/ + ; +/- ; +/+	10.50	8.00	3.13	2.38
+/+ ; +/+ ; +/-	10.50	10.00	3.13	2.98
+/+ ; -/- ; -/-	5.24	4.00	1.56	1.19
+/+ ; -/- ; +/+	5.24	5.00	1.56	1.49
+/+ ; +/+ ; -/-	5.24	2.00	1.56	0.60
+/+ ; +/+ ; +/+	5.24	4.00	1.56	1.19
**total larvae:**	**335.93**	**336**		**100**
