# Supplementary material for: The Gq/11 family of Gα subunits is necessary and sufficient for lower jaw development
Source: Development. 2025 Apr 17;152(8):dev204396. doi: 10.1242/dev.204396 (PMC12045641; doi:10.1242/dev.204396)
Supplement: Supplementary file 1 [file develop-152-204396_review_history.pdf]

The Gq/11 family of Gα subunits is necessary and sufficient for lower jaw development

Stanley M. Kanai, Chloe R. Garcia, MaCalia R. Augustus, Shujan A. Sharafeldeen, Elliott P. Brooks, Juliana Sucharov, Ezra S. Lencer, James T. Nichols and David E. Clouthier

DOI: 10.1242/dev.204396

Editor: Liz Robertson
Review timeline
Original submission: 16 September 2024
Editorial decision: 11 October 2024
First revision received: 18 February 2025
Accepted: 18 March 2025

Original submission

First decision letter

MS ID#: dev.204396

MS Title: The Gq/11 family of Gα subunits is necessary and sufficient for lower jaw development

Authors: Stanley Michinobu Kanai; Chloe R. Garcia; MaCalia R. Augustus; Shujan A. Sharafeldeen; Elliott P. Brooks; Juliana Sucharov; Ezra S. Lencer; James T. Nichols; David E. Clouthier
Article Type: Research Article

I have now received all the referees' reports on the above manuscript, and have reached a decision. The referees' comments are appended below, or you can access them online: please go to:

As you will see, the referees express considerable interest in your work, but have some significant criticisms and recommend a revision of your manuscript before we can consider publication. If you are able to revise the manuscript along the lines suggested, which may involve further experiments, I will be happy receive a revised version of the manuscript. Your revised paper will be re-reviewed by one or more of the original referees, and acceptance of your manuscript will depend on your addressing satisfactorily the reviewers' major concerns. Please also note that Development will normally permit only one round of major revision. If it would be helpful, you are welcome to contact us to discuss your revision in greater detail. Please send us a point-by-point response indicating your plans for addressing the referees' comments, and we will look over this and provide further guidance.

Please attend to all of the reviewers' comments and ensure that you clearly highlight all changes made in the revised manuscript. Please avoid using 'Tracked changes' in Word files as these are lost in PDF conversion. I should be grateful if you would also provide a point-by-point response detailing how you have dealt with the points raised by the reviewers in the 'Response to Reviewers' box. If you do not agree with any of their criticisms or suggestions please explain clearly why this is so.

Reviewer 1

Advance summary and potential significance to field

© 2025. Published by The Company of Biologists under the terms of the Creative Commons Attribution License (https://creativecommons.org/licenses/by/4.0/).

This is an excellent study that is well performed, convincing, and a pleasure to read. It addresses the mechanisms by which Edn1 signals to pattern the lower jaw, an important question as disruptions of this pathway cause lower jaw patterning defects, including in human syndromes. From mouse studies, it was unclear which G protein was required to transduce Edn1 signaling as conventional and tissue-specific knockout of Ga q/11 proteins resulted in only partial Edn1 phenocopy. Using zebrafish, the authors convincingly show that Ga11 protein transduces Edn1 signaling in zebrafish, using a combination of knockouts and gain-of-function approaches. The results are crystal clear, with both loss and gain of function phenotypes supporting a role of Ga11 in Edn1 signaling. The writing is also superb, including providing plausible explanations for the lack of severe Edn1-like phenotypes using P0-Cre to knockout Gaq in mouse and potential paralog switching to account for the greater importance of Ga11 in fish versus mouse.

Comments for the author

I feel like the manuscript could be published as is, though I have one suggestion for improvement. gnaq+/+; 11a-/-; 11b-/- is a critical genotype to make the argument that gnaq is dispensable and as such would benefit by being moved to the main Fig. 5. Also, while the image in Fig. S7C is convincing in showing an Edn1-like phenotype, its quality its poor compared to the images in Fig. 5. It would improve the paper to provide a better flat-mount image of this critical genotype, as well as quantitation as provided for the other genotypes in Fig. 5.

Reviewer 2

Advance summary and potential significance to field

In their manuscript "The Gq/11 family of Gα subunits is necessary and sufficient for lower jaw development," Kanai et al. use multiple approaches to show that Gq/11 is the downstream of Edn1/Ednra is patterning of the lower jaw. Using a chemical inhibitor and genetic approaches, they show loss of Gq/11 activity lead to phenotypes reminiscent of edn1 mutants, with the reduced expressivity of the chemical inhibitor mirroring partial loss of edn1 signaling while gq/11 triple mutants recapitulating full edn1 loss. They go on to show changes gna11a and b are necessary for lower jaw development while Gnaq appears dispensable for lower jaw formation yet sufficient for lower jaw and homeotic transformations of upper jaw to lower jaw in edn1 mutants. The authors' results will be of interest to the readership of Development, and the broader craniofacial research community. However, several major concerns exclude publication of the manuscript in its current form.

Comments for the author

Major Concerns:

1. The authors state that G14 also contains the same AAs that YM inhibition but never mention any interaction again. Given the lower expressivity of phenotypes in YM-treated larvae, the authors need to rule out that the impact of YM is not thought G14. This could be done by expression analysis of G14 in the arches.
2. It is not clear why the authors chose to make a new Edn1 mutant rather than use the original sucker mutant allele.
3. The authors need to explain the use of the sox10;fli1 double transgenic line for cell sorting CNCCs as non-zebrafish users may not understand why the double transgenic line is necessary.
4. The authors state on Fig4 that YM acts on Gq/11 and not other Gq/11 receptors, while there data suggests this they have not ruled out off target effects. It is possible that YM expressivity in edn1 hets is off target and simply additive to the reduction in edn1 signaling. The edn1 mutants are so severe in comparison you can rule out off target effects that are masked by the severity of the phenotypes. The authors need to strengthen the evidence for their statement or soften their stance.
5. Sanger sequencing figures for all mutants generated in this manuscript. The methods state that sanger sequencing was performed on backcrossed F1 adults yet the chromatographs show homozygosity of the indel where one would expect mixed signals between the WT and indel alleles in heterozygous fish. Was this intentional in the graphs or the actual outcome of the sequencing?

© 2025. Published by The Company of Biologists under the terms of the Creative Commons Attribution License (https://creativecommons.org/licenses/by/4.0/).

6. The authors state that Gnaq is dispensable for facial development using Figure 5H shows that gnaq mutant;gna1a/b hets and triple heterozygotes displayed similar phenotypes, yet both showed variation in phenotypic scoring while scoring of WT siblings was not shown. Without the WT scoring showing similar variation as the gnaq mutant;gna1a/b hets and triple heterozygotes, the authors cannot rule out that gnaq does not have a subtle impact on craniofacial development.
7. An alternative explanation is that gnaq is simply not expressed in arches 1 or 2. While the low expression of gnaq in the sequencing data suggests that it may be expressed in the CNCCs the authors need to perform in situ hybridization to show that it is actually express in cells that contribute to the 1st and 2nd arch CNCCs that give rise to the structures impacted in edn1 signaling.
8. Given that the authors state that gnaq is dispensable for jaw formation, the authors need to state why they chose to generate an overexpression construct.
9. In the discussion the authors state that their work with YM "showed that the solubility limit of YM prevented complete (sic) inhibition of Gq/11 activityâ€¦" Their work did not show the solubility limit but the toxic limit of DMSO. Was the stock concentration of YM in DMSO as high as it could go? Could they not concentration YM more in DMSO, there by decreasing the amount of DMSO volume needed?

Reviewer 3

Advance summary and potential significance to field

In the manuscript titled "The Gq/11 Family of Gα Subunits is Necessary and Sufficient for Lower Jaw Development," the authors demonstrate that the Gq/11 signaling pathway, acting downstream of Edn1/Ednra, is crucial for lower jaw development in zebrafish. The study provides strong in vivo evidence supporting this finding. The study is of high quality. There are some points that need to be addressed to further elucidate the mechanistic aspects of the conclusions.

Comments for the author

Major Points:
1. The discrepancy in phenotype observed with Gq/11 inhibition compared to edn1-/-mutants requires further discussion. In addition to directly validating the inhibition efficiency (see point 3 below), what is the in vivo expression pattern of Gq/11 during zebrafish head development? Is it more restricted than that of Edn1/Ednra and specifically associated with the mandible region?
2. The phenotypic analysis primarily uses whole-mount skeletal staining, which may miss milder defects in other craniofacial regions, particularly the maxillary area. A more comprehensive assessment—including histology, micro-CT imaging, and/or marker analysis—would offer a more thorough evaluation of craniofacial defects.
3. The authors should directly measure changes in Gq/11 expression levels or activity following YM-254890 treatment and in the gnaq-/-, gna11a-/-, and gna11b-/- zebrafish knockout models. Restoration of Gq/11 expression levels or activity should also be measured in the hsp70l:Gq-Q209L rescue experiment.
4. The new mechanism discovered in this study needs to be clearly stated. Single-cell RNA-seq analysis could be a valuable tool to identify detailed mechanisms. Please specify which cell populations are associated with the defects observed after YM-254890 treatment and what specific molecular changes are involved in these populations. More importantly, how do molecular changes in these populations lead to alterations in cell fate and bone shape? Do they affect cell proliferation, differentiation, apoptosis, or migration, resulting in the observed bone shape changes?

Minor Points:
1. Missing period at the end of the third paragraph in the introduction.
2. Abbreviations are not explained in the figure legend of Figure 4.

First revision

© 2025. Published by The Company of Biologists under the terms of the Creative Commons Attribution License (https://creativecommons.org/licenses/by/4.0/).

Author response to reviewers' comments

Reviewer 1 Comments for the Author:
1. I feel like the manuscript could be published as is, though I have one suggestion for improvement. gnaq+/+; 11a-/-; 11b-/- is a critical genotype to make the argument that gnaq is dispensable and as such would benefit by being moved to the main Fig. 5. Also, while the image in Fig. S7C is convincing in showing an Edn1-like phenotype, its quality its poor compared to the images in Fig. 5. It would improve the paper to provide a better flat-mount image of this critical genotype, as well as quantitation as provided for the other genotypes in Fig. 5.

We have added a high-quality image of a flat-mount for a *gnaq*$^{+/+}$; *gna11a*$^{-/-}$;*gna11b*$^{-/-}$ larvae as Figure 5Cand have also included quantitative analysis of this genotype in Figure 5J. In doing so, Panel C was removed from Fig. S9 (previously Fig. S7)

Reviewer 2 Comments for the Author:
1. The authors state that G14 also contains the same AAs that YM inhibition but never mention any interaction again. Given the lower expressivity of phenotypes in YM-treated larvae, the authors need to rule out that the impact of YM is not thought G14. This could be done by expression analysis of G14 in the arches.

We state in the Results section (Pg. 8) that "Other Gq/11 family members, including *gna14, gna14a* and *gna15.1-15.4,* were not considered because they exhibit cell-type specific expression in sensory organs and hematopoietic lineages (Ohmoto et al., 2011; Oka and Korsching, 2011)." We have also added a new paragraph in the Discussion section elaborating on the data ruling out the requirements of G14 and G15 in craniofacial development (Pg. 10): "Our genetic analysis with mutant alleles has revealed differential requirements for Gq/11 genes in zebrafish craniofacial development. Of the four members of the Gq/11 gene family – Gq, G11, G14, and G15 – we generated mutant alleles for Gq (*gnaq*) and G11 (*gna11a* and *gna11b*) only. Larvae that are homozygous mutants for both G11 genes (*gnaq*$^{+/+}$; *gna11a*$^{-/-}$; *gna11b*$^{-/-}$) recapitulated the *edn1*$^{-/-}$ mutant phenotype (Fig. 5C), suggesting that G11 genes are required for lower jaw patterning, while the Gq, G14, and G15 genes are not. The dispensability of G14 and G15 in lower jaw patterning is consistent with studies that have reported the expression of G14 and G15 genes are restricted to sensory organs and hematopoietic lineages (Ohmoto et al., 2011; Oka and Korsching, 2011)."

Furthermore, our scRNA-seq data indicate that genes encoding for G14 (*gna14* and *gna14a*) are not detectable in the first and second pharyngeal arches clusters. Given the difficulty in detecting *gnaq* and *gna11a* with either ISH (See new Fig. S8) or qPCR (See new Fig. S7), we did not proceed with ISH for G14 genes.

2. It is not clear why the authors chose to make a new Edn1 mutant rather than use the original sucker mutant allele.

The original *sucker* allele causes an amino acid substitution (p.Asp8Val) in the bioactive portion of Edn1. While in vitro studies have reported that Edn1 p.Asp8Val peptides exhibit 125-fold less activity than wild-type Edn1 (Nakajima 1989, Watanabe 1991), whether the Edn1 p.Asp8Val peptides result in partial or complete loss of activity in vivo was unknown. Therefore, it was unknown whether the *sucker* phenotype represented a partial or complete loss of Edn1 activity. Our new *edn1* allele (co3009), which ablates nearly the entire coding sequence for the bioactive portion of Edn1, recapitulated the *sucker* craniofacial phenotype (Fig. S1). This result confirmed that the *sucker* craniofacial phenotype is caused by a complete loss of Edn1 activity. Using this new allele ensured that we were comparing our Gq/11 mutant phenotypes with a proven *edn1*-null phenotype. We have added this explanation to the Materials and Methods section.

3. The authors need to explain the use of the sox10;fli1 double transgenic line for cell sorting CNCCs as non-zebrafish users may not understand why the double transgenic line is necessary.

© 2025. Published by The Company of Biologists under the terms of the Creative Commons Attribution License (https://creativecommons.org/licenses/by/4.0/).

We have added an explanation as to why we are using *sox10-RFP;fli-GFP* double transgenic line to cell sort neural crest cells for subsequent scRNA-sequencing in the Results section (Pg 6): "Fluorescence-activated cell sorting (FACS) of *sox10:mRFP;fli1a:EGFP* double-positive cells allows us to purify cranial NCC populations in zebrafish embryos (Askary 2017, Mitchell 2021)."

4. The authors state on Fig4 that YM acts on Gq/11 and not other Gq/11 receptors, while there data suggests this they have not ruled out off target effects. It is possible that YM expressivity in edn1 hets is off target and simply additive to the reduction in edn1 signaling. The edn1 mutants are so severe in comparison you can rule out off target effects that are masked by the severity of the phenotypes. The authors need to strengthen the evidence for their statement or soften their stance.

The reviewer is correct that we cannot completely rule out that the increased severity of phenotypes in *edn1* heterozygous embryos is caused by YM acting on other Gq/11-coupled receptors. We have addressed this concern by removing the following text in the Results section: "..indicating that YM is specifically acting on Gq/11 downstream of Ednra and not on other Gq/11-couple receptors." (Pg. 8).

5. Sanger sequencing figures for all mutants generated in this manuscript. The methods state that sanger sequencing was performed on backcrossed F1 adults yet the chromatographs show homozygosity of the indel where one would expect mixed signals between the WT and indel alleles in heterozygous fish. Was this intentional in the graphs or the actual outcome of the sequencing?

The Sanger sequencing traces shown in Fig. S9 (previously Fig. S7) are from homozygous mutant animals. This description has been added to the figure legend for clarity.

6. The authors state that Gnaq is dispensable for facial development using Figure 5H shows that gnaq mutant;gna1a/b hets and triple heterozygotes displayed similar phenotypes, yet both showed variation in phenotypic scoring while scoring of WT siblings was not shown. Without the WT scoring showing similar variation as the gnaq mutant;gna1a/b hets and triple heterozygotes, the authors cannot rule out that gnaq does not have a subtle impact on craniofacial development.

As requested by the reviewer, we have added the quantified phenotype score for wild-type larvae, which shows an average phenotype score of 0 with no variation (Fig. 5J).

We agree with the reviewer that the data as shown does not rule out the possibility that *gnaq* makes subtle contributions to craniofacial development. At the same time, however, this possibility is not supported by the data. If *gnaq* is having a subtle impact on craniofacial development, then we would expect variation of phenotype score to be smaller in $q^{+/-}$; $11a^{+/-}$; $11b^{+/-}$ larvae relative to $q^{-/-}$; $11a^{+/-}$; $11b^{+/-}$ larvae (because phenotype scores will be overall lower in $q^{+/-}$; $11a^{+/-}$; $11b^{+/-}$ larvae). We would also expect phenotype scores to be lower in $q^{+/-}$; $11a^{+/-}$; $11b^{-/-}$ larvae relative to $q^{-/-}$; $11a^{+/-}$; $11b^{-/-}$ larvae, and similarly be lower in $q^{+/-}$; $11a^{-/-}$; $11b^{+/-}$ larvae relative to $q^{-/-}$; $11a^{-/-}$; $11b^{+/-}$ larvae. However, these differences are not observed, and the lack of statistical significance between these genotypes have been updated in Fig. 5J. To strengthen our conclusion that *gnaq* is dispensable for lower jaw development, we have also added the flat-mount and quantified phenotype score for $q^{+/+}$; $11a^{-/-}$; $11b^{-/-}$ larvae (new Fig. 5C,J), which phenocopies *edn1*$^{-/-}$ mutants with full penetrance.

With that said, we still agree with the reviewer that we cannot rule out very subtle contributions of *gnaq* to craniofacial structures that would be uncovered by exhaustive morphometric analysis and have noted this in the Discussion (Pg. 11): "One possible explanation for this discrepancy is that *gnaq* does in fact contribute to lower jaw patterning in zebrafish albeit to a lesser extent, with the loss of *gnaq* resulting in skeletal defects that are imperceptible with our current analysis.".

7. An alternative explanation is that gnaq is simply not expressed in arches 1 or 2. While the low expression of gnaq in the sequencing data suggests that it may be expressed in the CNCCs the

© 2025. Published by The Company of Biologists under the terms of the Creative Commons Attribution License (https://creativecommons.org/licenses/by/4.0/).

authors need to perform in situ hybridization to show that it is actually express in cells that contribute to the 1st and 2nd arch CNCCs that give rise to the structures impacted in edn1 signaling.

In our original discussion, we implied but did not explicitly state that *gnaq* is dispensable for lower jaw patterning because it is simply not expressed in cranial NCCs. The text has been revised for clarity in the Discussion (Pg. 11): “One possible explanation for this discrepancy is that *gnaq* does in fact contribute to lower jaw patterning in zebrafish albeit to a lesser extent, with the loss of *gnaq* resulting in skeletal defects that are imperceptible with our current analysis. An alternative explanation is that *gnaq* is simply not expressed in the lower jaw progenitor cells and therefore does not contribute to lower jaw patterning in zebrafish. The latter explanation is consistent with our gene expression analysis using scRNA-seq (Fig. S6), qPCR (Fig. S7), and ISH (Fig. S8), which suggest that *gnaq* expression is absent or lower relative to *gna11a* and *gna11b* in cranial NCCs (Fig. S6-8)”.

We have also addressed the reviewer’s concern about the expression pattern of Gq/11 genes in the facial mesenchyme. We have performed in situ hybridization on wild-type embryos at 28 hpf using sense and antisense probes for *gnaq*, *gna11a*, and *gna11b* (Fig. S8). Our analysis of *gnaq* and *gna11a* was inconclusive, as we did not observe differences between sense and antisense probes. However, we found that *gna11b* is broadly expressed in the head mesenchyme, including the pharyngeal arches. We believe that *gnaq* and *gna11a* are difficult to detect at this stage of development due to their low expression levels. This is supported by our qPCR analysis of the head, which showed that expression levels for *gnaq* and *gna11a* were lower relative to expression levels of *gna11b* (Fig. S7). While a more sensitive detection method such as RNAscope or Hybridization Chain Reaction (HCR) ISH may be more successful, time constraints have pushed this experiment into another study.

8. Given that the authors state that gnaq is dispensable for jaw formation, the authors need to state why they chose to generate an overexpression construct.

The proteins encoded by *Gnaq/gnaq* and *Gna11/gna11a/b* are functionally redundant (Malbon, 2005; Wettschureck and Offermanns, 2005). Furthermore, constitutively active mutants Gq-Q209L and G11-Q209L activate the same signaling pathways (Annala 2019; Lapadula 2019). We chose Gq-Q209L because we used this construct in previous studies and it was already in hand.

We had stated the functional redundancy of Gq and G11 in the Results (Pg. 9), but we have modified it to improve clarity. It now states: “Gq and G11 proteins are functionally redundant (Malbon, 2005; Wettschureck and Offermanns, 2005), and the constitutively active mutants Gq-Q209L and G11-Q209L activate the same signaling pathways in the context of uveal melanoma (Annala 2019; Lapadula 2019).”

9. In the discussion the authors state that their work with YM "showed that the solubility limit of YM prevented complete (sic) inhibition of Gq/11 activity..." Their work did not show the solubility limit but the toxic limit of DMSO. Was the stock concentration of YM in DMSO as high as it could go? Could they not concentration YM more in DMSO, there by decreasing the amount of DMSO volume needed?

The technical datasheet for YM-254890 states that the solubility limit in DMSO is 10 mM (9.6 mg/ml). Thus, we used a stock concentration of 10 mM YM-254890. For zebrafish embryo media, 1% DMSO is the upper limit that does not cause developmental defects (Hoyberghs, et al. 2021). To ensure that we do not exceed 1% DMSO in the embryo media, 100 uM YM-254890 was the highest concentration we could use. We have clarified this point in the Material and Methods section by including the previous two sentences (Pg. 16). We have also clarified the DMSO concentration limit that does not cause development defects in the Results section (Pg 5), and we also included in the discussion the fact that we cannot use a higher concentration than 100 uM while removing the statement concerning the “solubility limit of YM” (Pg 12).

Reviewer 3 Comments for the Author:

© 2025. Published by The Company of Biologists under the terms of the Creative Commons Attribution License (https://creativecommons.org/licenses/by/4.0/).

1. The discrepancy in phenotype observed with Gq/11 inhibition compared to edn1-/-mutants requires further discussion. In addition to directly validating the inhibition efficiency (see point 3 below), what is the in vivo expression pattern of Gq/11 during zebrafish head development? Is it more restricted than that of Edn1/Ednra and specifically associated with the mandible region?

The basis for the phenotypic discrepancy between YM-treated embryos and *edn1*$^{-/-}$ embryos is discussed in the Results (Pg. 7) and Discussion (Pg. 12) sections. Briefly, in the complete absence of Edn1/Ednra signaling, all lower jaw patterning genes are not expressed, and in turn all lower jaw structures fail to form. When Edn1/Ednra signaling is only partially lost, a subset of lower jaw patterning genes are expressed, and a subset of lower jaw structures form (Walker 2006, Miller 2007, Walker 2007, Sato 2008a, Ruest and Clouthier 2009, Tavares 2012). YM-treated embryos elicit phenotypes (Fig. 2) and gene expression changes (Fig. 3) that resemble partial loss of the Edn1/Ednra signaling pathway. Therefore, we concluded that the phenotype observed with Gq/11 inhibition (YM treatment) represents a partial loss of Edn1/Ednra signaling, as opposed to a complete loss of Edn1/Ednra signaling (e.g. *edn1*$^{-/-}$ mutants).

Determining the inhibition efficiency of YM in vivo is technically challenging and is beyond the scope of this study. However, we are currently developing a technique to directly measure Gq/11 activity in vivo and will be part of a future study. Regardless, the selectivity of YM for Gq/11 proteins has been extensively characterized (Takasaki et al., 2004; Nishimura et al., 2010; Schrage et al., 2015; Onken et al., 2018), forming the basis of our supposition that treating zebrafish embryos with YM will selectively target and inhibit Gq/11 proteins (Fig. 1).

The expression pattern of Gq/11 genes in zebrafish head development is not fully characterized. Our scRNA-seq data suggest that *gna11a* and *gna11b* are expressed in cranial NCCs, while *gnaq* is not (Fig. S6). Both *gna11a* and *gna11b* are broadly expressed in cranial NCCs, including the lower jaw progenitors, and overlaps with *ednraa* and *ednrab* expression (Nair 2007, Barske 2016). To further characterize expression patterns, we have performed additional analyses with qPCR (Fig. S7) and ISH (Fig. S8). Our qPCR analysis of the whole zebrafish head at 28 hpf shows that *gna11b* is expressed at levels comparable to *dlx2a,* whereas *gnaq* and *gna11a* expression levels are relatively lower (Fig. S7). *dlx2a* was used as a reference because it is a well-characterized gene that is highly expressed in the forebrain and the pharyngeal arches (Fig. S8). We then performed whole mount ISH on 28 hpf zebrafish embryos and analyzed coronal sections through the head. Of the three Gq/11 genes tested, only *gna11b* was detectable by ISH, showing broad expression in the head, including the pharyngeal arches. Given the low expression levels of the Gq/11 genes (and G protein genes in general), it will be necessary to use a more sensitive detection method like RNAscope or Hybridization Chain Reaction ISH to comprehensively characterize expression patterns in a future study.

We have added text in the Results section (Pg. 8) referencing Fig. S7 and S8. The Materials and Methods section has also been updated with experimental details.

2. The phenotypic analysis primarily uses whole-mount skeletal staining, which may miss milder defects in other craniofacial regions, particularly the maxillary area. A more comprehensive assessment—including histology, micro-CT imaging, and/or marker analysis—would offer a more thorough evaluation of craniofacial defects.

While microCT could provide metrics on subtle changes, the purpose of our study was to compare gross skeletal changes in Gq/11 mutants with existing data on skeletal changes that occur following disruption of Edn1 activity. In the future, we could use microCT to re-evaluate both these existing models and our new models to examine how small changes in signaling affect phenotypic variation. Note that to date, changes in maxillary structures have not been identified in loss-of-function mutants of endothelin pathway members in the mouse, zebrafish or humans.

3. The authors should directly measure changes in Gq/11 expression levels or activity following YM-254890 treatment and in the gnaq-/-, gna11a-/-, and gna11b-/- zebrafish knockout models. Restoration of Gq/11 expression levels or activity should also be measured in the hsp70l:Gq-Q209L rescue experiment.

© 2025. Published by The Company of Biologists under the terms of the Creative Commons Attribution License (https://creativecommons.org/licenses/by/4.0/).

Gq/11 expression levels do not change following YM-254890 based on our scRNA-seq data. We agree that it is important to assess changes in endogenous Gq/11 activity after perturbation, though there are significant challenges in measuring changes in endogenous Gq/11 activity in vivo in any setting. As part of our follow-up studies, we are developing in vivo tools to measure changes in intracellular Ca^{2+} levels as a readout for Gq/11 activity. However, we can indirectly measure changes in Gq/11 activity when using an over-expression approach. One readout of Gq/11 activity is ERK phosphorylation through the MAP Kinase pathway (Van Raamsdonk 2009). For the hsp70l:Gq-Q209L rescue experiment, we used immunohistochemistry on sectioned sibling control and hsp70l:Gq-Q209L zebrafish embryos to detect phosphorylated ERK. We now show in Fig. S11 that ERK phosphorylation is dramatically increased in the ectoderm and the pharyngeal arch mesenchyme, including cranial NCCs, 3 hours following heat shock. From these findings, we can infer that the Gq-Q209L transgene is expressed in the neural crest and is likely the basis of the phenotypic rescue.

We have added text in the Results section (Pg 9) referencing Fig. S11. The Materials and methods section has also been updated with experimental details.

4. The new mechanism discovered in this study needs to be clearly stated. Single-cell RNA-seq analysis could be a valuable tool to identify detailed mechanisms. Please specify which cell populations are associated with the defects observed after YM-254890 treatment and what specific molecular changes are involved in these populations. More importantly, how do molecular changes in these populations lead to alterations in cell fate and bone shape? Do they affect cell proliferation, differentiation, apoptosis, or migration, resulting in the observed bone shape changes?

On pages 6 and 7, we stated that YM treatment specifically affected six out of eleven clusters – frontonasal, ventral arches 1 and 2, dorsal arch 1, dorsal arch 2, posterior arches 3-7 and cluster 5. We further stated that the changes observed specifically affect endothelin-pathway genes, including *dlx4a* and *dlx4b,* while also upregulating upper jaw-associated patterning genes such as *hey1* and *nr2f5*. It is well-established that this gene expression signature underscores the mechanism for the endothelin-mutant phenotype. In mouse, zebrafish and humans, the *Dlx4* gene is required for the formation of the intermediate and ventral first arch domains, which gives rise to Meckel's cartilage and the jaw joint in fish, and the mandible, jaw joint and some middle ear structures in mammals. We only briefly touched on these aspects because the cellular consequences of loss of the endothelin pathway have been extensively reported. Changes have also been reported in cell proliferation and apoptosis (but not in migration), though we have not discussed these aspects because it is not the focus of this manuscript. In short, the loss of endothelin signaling and its downstream target genes disrupts the patterning and subsequent differentiation of neural crest cells in the intermediate and ventral domains of the first and second arches. Again, these changes have been well-described and discussed in the literature.

Minor Points:
1. Missing period at the end of the third paragraph in the introduction.

We have added the missing period.

2. Abbreviations are not explained in the figure legend of Figure 4.

We have defined abbreviations in the figure legend for all figures including Fig. 4.

<u>Second decision letter</u>

MS ID#: dev.204396R1

MS Title: The Gq/11 family of Gα subunits is necessary and sufficient for lower jaw development

© 2025. Published by The Company of Biologists under the terms of the Creative Commons Attribution License (https://creativecommons.org/licenses/by/4.0/).

Authors: Stanley Michinobu Kanai; Chloe R. Garcia; MaCalia R. Augustus; Shujan A. Sharafeldeen; Elliott P. Brooks; Juliana Sucharov; Ezra S. Lencer; James T. Nichols; David E. Clouthier
Article Type: Research Article

I am happy to tell you that your manuscript has been accepted for publication in Development, pending our standard publication integrity checks.

Reviewer 1

The authors have addressed my only initial minor concern. I have also read the responses to the issues raised by the other two reviewers and feel that the authors have comprehensively addressed all concerns in a rigorous and thoughtful way.

Reviewer 2

The authors' have addressed all concerns of this reviewer and the results will be of interest to the readership of Development, and the broader craniofacial research community.

Reviewer 3

All my concerns have been addressed. Nice job.

© 2025. Published by The Company of Biologists under the terms of the Creative Commons Attribution License (https://creativecommons.org/licenses/by/4.0/).
